# Numerical simulation and theoretical study on the impact of wind-sand flow of high-speed trains in long tunnel space

**DOI:** 10.1038/s41598-024-64481-y

**Published:** 2024-06-13

**Authors:** Shiqun Li, Sihui Dong, Yonghua Li, Liping Zhou

**Affiliations:** 1 College of Locomotive and Rolling Stock Engineering, Dalian Jiaotong University, Liaoning, 116028 Dalian China; 2 School of Traffic and Transportation Engineering, Dalian Jiaotong University, Liaoning, 116028 Dalian China

**Keywords:** HST; 3D, DDES; High wind, Sand, Long tunnel infrastructure (HWS, LT); Flow and heat transfer; Correlation models, Engineering, Civil engineering, Mechanical engineering, Fluid dynamics

## Abstract

When high-speed trains (HST) run in enclosed spaces such as long tunnels, the thermal accumulation of their suspension devices is continuous and cannot be effectively dissipated. In addition, previous experiments or simulations for the heat dissipation of HST in tunnel spaces did not consider the impact of sand. To clarify the impact of HWS-LT on the heat accumulation of HST equipment cabin, this study used the CFD method to numerically simulate the impact of different wind-sand flow concentrations or no-sand wind on the cooling of equipment in the long tunnel space. Firstly, the sand particles in the wind-sand flow gather at the tunnel entrance and enter the equipment cabin with the train as it enters the tunnel. This boundary condition is more in line with actual engineering situations. Secondly, both flows show asymmetric intrusion into the cabin due to the asymmetrical tunnel arrangement, but the sand particles in the wind-sand flow are affected by the vortices and tunnel walls, resulting in more asymmetric flow and some particles being trapped in the grids or filters, leading to outflow *ρ*_*Q*_ < inflow *ρ*_*Q*_*.* Under the wind-sand flow condition, the temperature of some equipment surfaces shows more significant increases than under the no-sand wind. Finally, contrary to popular perception, the wind-sand flow carrying sand particles can dissipate heat more effectively than no-sand wind, and the higher the volume fraction *φ* within a certain concentration range, the better the heat dissipation effect. This is because the wind-sand flow has a higher specific heat capacity, which can remove some heat from the contact point between the sand particles and the equipment wall upon contact. The higher sand particle concentration increases the contact frequency and contact area between the sand particles and the equipment wall, and the heat transfer pathway and heat dissipation efficiency are improved.

## Introduction

The high speed of HST requires a more powerful traction and drive system, which in turn requires a more appropriate thermal system design^1^. Inefficient cooling can lead to equipment performance degradation, even threatening the safety of equipment operation. Therefore, to ensure safe operation and performance improvement of HST, the heat dissipation of equipment in the equipment cabin is still an important research topic^2,3^.

The natural ventilation and heat dissipation performance of train suspension equipment have received widespread attention from researchers since the extensive operation of HST. Huang et al.^4^ compared the scales and patterns of temperature changes in the equipment cabin under different environments; Zhang et al.^5^ discussed the influence of grille position and shape on cooling performance. The methods used for these issues are mainly wind tunnel tests^6^ and CFD simulations. Previous studies generally believed that compared with experimental methods, numerical simulation is easier to reproduce the operation process of trains in complex scenarios, and has a larger transient aerodynamic data volume and more detailed three-dimensional flow information^7–10^. In addition, researchers have also paid attention to the heat dissipation issues in special scenarios, among which the enclosed space in tunnels is considered more worthy of attention. Due to the narrow and enclosed environment of the tunnel^11^, the repeated transmission of pressure waves in the tunnel not only causes the aerodynamic force acting on the train body to change dramatically^12^, but also makes the air flow near the train complex and difficult to predict, affecting the ventilation and heat dissipation of the equipment cabin. However, previous experiments or simulations on heat dissipation in tunnel spaces did not consider the influence of sand particles. The natural wind that cools the train suspension equipment was considered to be composed of a single gas phase. However, in long tunnels in high-temperature sandy areas, there are often high concentrations of sand particles and dust, and the natural wind will drive the sand particles to form a wind-sand flow. Over time, a certain concentration of sand particles will hang or deposit in the tunnel. The impact and mechanism of the wind-sand flow on the heat dissipation of the suspension equipment in the tunnel are unknown. Previous studies on this level are relatively conservative, and there is a lack of attention to the "high wind-sand-long tunnel" infrastructure scenario.

The regions of North America, Central Africa, Australia, and Central Asia are severely desertified. In particular, in China^13–15^, the continuous mountainous terrain in the northwest is more common, and the terrain of railway trunk lines is more complex. The infrastructure scenario composed of a long tunnel and a high temperature and high wind-sand environment has become more and more common in high-speed railways (HSRs), and the mainline tunnel of the Lanxin Railway has 64 tunnels with a total length of 187.16 km, accounting for 10.5% of the total length of the railway. The tunnel in the wind zone is located in a high wind-sand area, and the highest temperature can reach 43.5℃. Due to the long-term effects of external high-temperature, high-concentration, and high-velocity wind and sand on the tunnel, the sand concentration is high. In addition, due to the relatively enclosed space inside the tunnel and the relative stability of air flow, there is a serious sand deposition phenomenon inside the tunnel. Therefore, when HST travels through the infrastructure scenario composed of "high wind-sand-long tunnel" (hereinafter referred to as "HWS-LT" scenario), the train may face higher safety risks than no-sand wind for the following reasons^16–18^:

- (1) After the long tunnel located in a high wind-sand area is put into operation, due to the narrow channel effect caused by the mountain blocking, the tunnel space is inevitably affected by the external strong wind and sand for a long time. The tunnel is filled with high-concentration wind and sand flow, and even sand deposition trajectory phenomena may appear with the change of time^19^.
- (2) Due to the fact that the longitudinal length of the tunnel in the sandy area is usually greater than 1 km, the HST will travel in a limited space for a long time after entering the tunnel. As the travel time increases, the heat generation of its suspension equipment continues to increase, and due to the narrow and long design of the tunnel, the dynamic interaction between compression waves, expansion waves and asymmetric vortices caused by the blocking effect, wind and sand particles invade the equipment cabin with complex movement rules. While the grille in the cabin effectively reduces the sand concentration, the heat dissipation performance cannot be guaranteed. The local recirculation area in the cabin accumulates heat rapidly and cannot be effectively dissipated, and the contradiction between "heat dissipation and dust removal" cannot be effectively solved^20,21^.

In summary, this study focuses on the poor heat dissipation of HST equipment in the HWS-LT scenario and uses the CFD simulation software Fluent for analysis and simulation. Firstly, a long tunnel located in the "Qijiaojing" wind-prone area on the second double line of the Lanxin Railway was selected as the object of study. Various geometric and numerical models were determined, and the 3D-DDES model was used instead of the traditional LES standard *k*-*ε* turbulence model, which has better mixing-phase simulation performance^22,23^. Secondly, in order to better analyze the impact mechanism and differences of sand-free wind and sand flow on equipment heat dissipation efficiency in a tunnel environment, CFD simulation and analysis were carried out for the flow characteristics of millimeter-scale sand flow under multi-field coupling, particle dispersion characteristics, and transient airflow effects. Based on this, the heat dissipation mechanism of wind-sand flow (including sand-free wind) under different sand particle concentration gradients was studied, and the "mixed-phase shock device block" scenario was established to further explore the mechanism of single-device heat transfer under the influence of wind-sand flow under multiple variables. Finally, based on the actual situation of HST traveling in tunnels in a wind sand area, an evaluation model applicable to sand and wind for heat dissipation efficiency was established, which realizes the pre-evaluation of heat dissipation of the "environment-heat source" system and provides a theoretical basis for the design and optimization of heat-generating equipment. The related terms involved in this paper are shown in Nomenclature.

## Methodology

**Figure 1:**
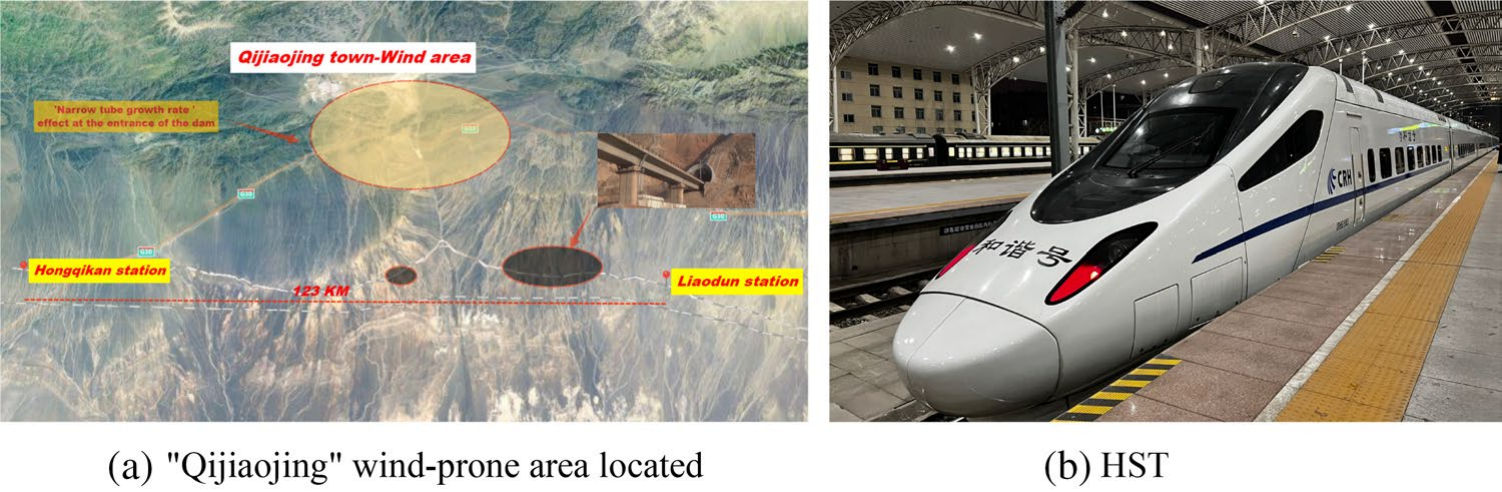
Model selection. (**a**) “Qijiaojing” wind-prone area located, (**b**) HST.

The second double line of the Lanxin Railway passes through four major wind-prone areas^18^, one of which is the "Qijiaojing" wind-prone area located in Qijiaojing Town, with a total length of 123 km and a low altitude and narrow valley mouth, forming a "The effect of narrow" effect, resulting in a strong wind force containing a large amount of sand and stones, creating a harsh operating environment for HST, as shown in Fig. 1a. The topography distribution in Fig. 1a of this study was obtained from the map engine developed independently by Baidu Company: Baidu Maps (https://map.baidu.com), and complies with any copyright requirements emphasized by Baidu Company (https://map.baidu.com/zt/client/copyrightPc/index.html). In order to clarify the impact of the HWS-LT scenario on the heat accumulation in the HST equipment cabin, this study takes the second double line of the Lanxin Railway in China as the research context and the CRH5G train with the latest organization as the research object, as shown in Fig. 1b.

### Geometry model and computational domain

**Figure 2:**
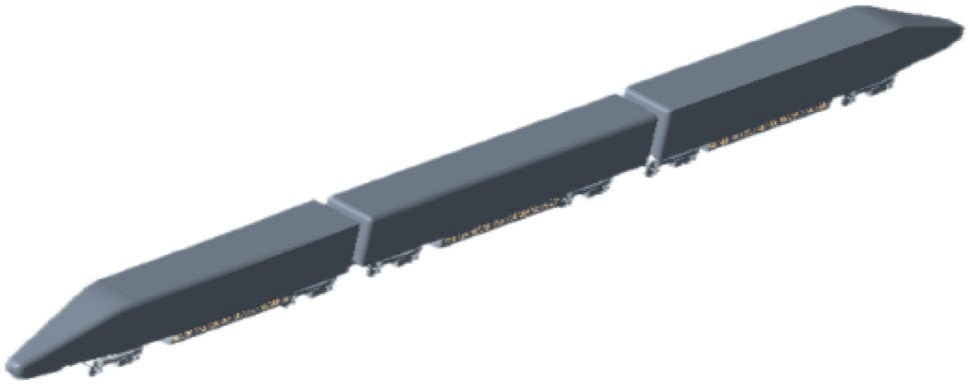
Geometric model.

In order to solve the continuity problem, we simplified the entire train set. According to the limit length proposed by Cooper, within a certain length downstream from the front of the train (less than the length of one train of carriages), the flow field structure almost no longer changes. Shortening the length of the train does not change the essential characteristics of the wind-sand flow. This design is similar to the design used by Gil et al. and Muld et al.^24,25^, which is considered to be capable of correctly reproducing the aerodynamics of high-speed trains. Therefore, in this study, the 3D model adopts a connected structure of "front car-middle car-rear car". All details less than the size of the surface element are not considered, and single elements such as the pantographs are omitted. The length (*L*) of the train model is 81.8 m, the height (*H*) is 3.93 m, and the width (*W*) is 3.09 m, with a cross-sectional area (*S*) of approximately 11.5 m^2^. The 3D model of the train is shown in Fig. 2.

**Figure 3:**
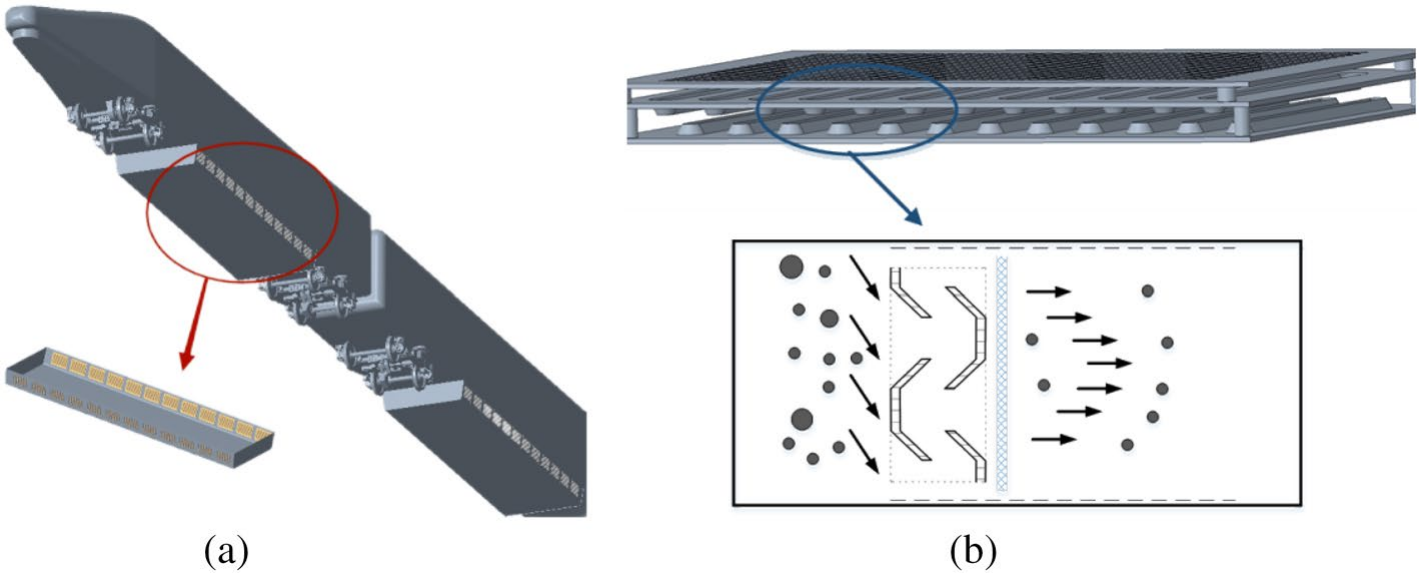
Train bottom assembly drawing and grille layout.

Hu et al.^26^ conducted a numerical study on the suspension equipment cabin of different carriages and found that the heat accumulation in the equipment cabin of the rear carriage is the worst. In order to save computational resources, the main heat source object of this study is the suspended equipment at the bottom of the rear carriage of HST, and a calculation model of the equipment cabin is established. The equipment cabin calculation model is simplified according to the original assembly of the train, and for the equipment and equipment brackets, their basic appearance shapes are retained. Paz et al.'s study showed that the maximum flow rate of the bottom cold air fan is 1094 m^3^/h, which is very small compared to the overall ventilation in the cabin and does not significantly affect the overall heat distribution in the cabin^12^. In addition, the original model's grille-style ventilation openings are retained for the equipment cabin ventilation openings, and the grilles are installed according to the original positions. The grille model used in this study is a V-V wind and sand barrier grille, with a filter screen and sand leakage holes. The bottom installation diagram and grille layout are shown in Fig. 3.

**Figure 4:**
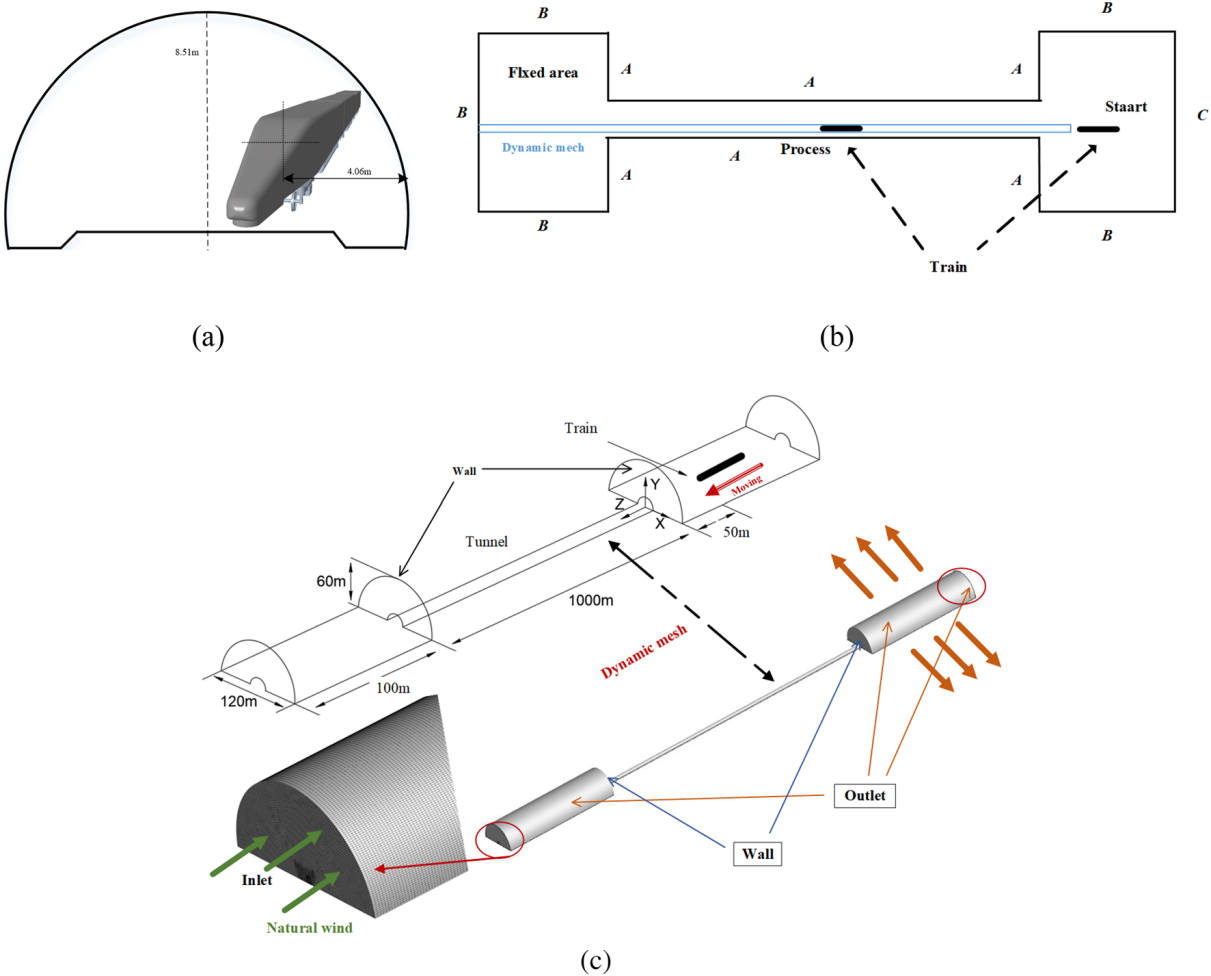
Schematic diagram of tunnel section; calculation domain diagram; boundary conditions.

The selected tunnel model is a complex line long tunnel of the second double line of the Lanxin Railway, with an actual length of about 2.5 km. To save computational resources, the length (*L*_*t*_) of the tunnel calculation model is 1 km, and the width (*W*_*t*_) and height (*H*_*t*_) are approximately 13.28 m and 8.51 m, respectively. The cross-sectional area of the tunnel is 100 m^2^, and the blockage ratio beta is approximately 11.5%. In addition, in order to restore the external open wind-sand environment and facilitate the analysis of wind-sand movement In the external environment, a semicircular flow field area with a diameter of 120 m and a length of 100 m are set at both ends of the tunnel. The entire calculation domain is 1200 m long, 120 m wide, and 60 m high. The initial position of the train is 50 m away from the entrance of the tunnel. Figure 4 shows a schematic diagram of the calculation domain and boundary conditions.

Some details, such as rail plates and tracks, are omitted from the calculation to save resources, and comparison with the corresponding model experiment results shows that the omission is reliable^5^. It is worth noting that although the length of the tunnel model is only 40% of the actual prototype, the one-dimensional characteristics of the pressure distribution in the tunnel have been extensively studied, and the distribution of wind-sand particles in the tunnel is closely related to the pressure distribution. Therefore, this simplification has little effect on the analysis of flow characteristics and heat dissipation mechanisms in equipment cabin during high-speed train operation^27,28^.

### Boundary conditions and Meshing strategy

The CFD simulation in this study is divided into two parts. First, to evaluate the wind erosion in a long tunnel under high wind and sand conditions, in order to set boundary conditions that are more in line with engineering practice, steady-state calculations are performed on the exterior open environment-tunnel space without trains. Sand particles and natural wind are released horizontally from the inlet until the wind and sand fill the tunnel and stabilize. Second, based on the wind and sand environment under steady-state conditions, numerical simulations of HST trains traveling inside the tunnel are conducted using dynamic mesh layering technology. The dynamic mesh is not covering the entire calculation domain, and Fig. 4b shows the division between the sliding area and the fixed area. The interface between the two areas is used for data transfer between meshes. Figure 4c shows the mesh partitioning area and flow boundary conditions, in which the ground and mountain walls are set as Wall; In order to simulate the important behavior of particles on the Earth's surface, the longitudinal and tangential restitution coefficients of the wall surfaces in the fluid domain, mountain walls, surfaces inside equipment cabin, and grids and filter screens are set to 0.92^29^. The inlet boundary (C) of the flow field domain is set as a velocity inlet, with a temperature of 309.15 K and a blackness value of 1. The outer boundary (B) is set as a pressure outlet, with a backflow temperature of 309.15 K and a blackness value of 1. The heat flux density of the heating equipment is given, where *Q*_*1*_ represents the heat flux density of the auxiliary converter, which is 480W/m^2^; *Q*_*2*_ represents the heat flux density of the air conditioning condenser, which is 110W/m^2^; *Q*_3_ represents the heat flux density of the traction converter, which is 350W/m^2^. The blackness of the heating equipment walls is uniformly set to 0.35, and the material is Steel. C is also set as a sand release surface, and the physical properties of the multiphase flow are compressible steady flow.

**Figure 5:**
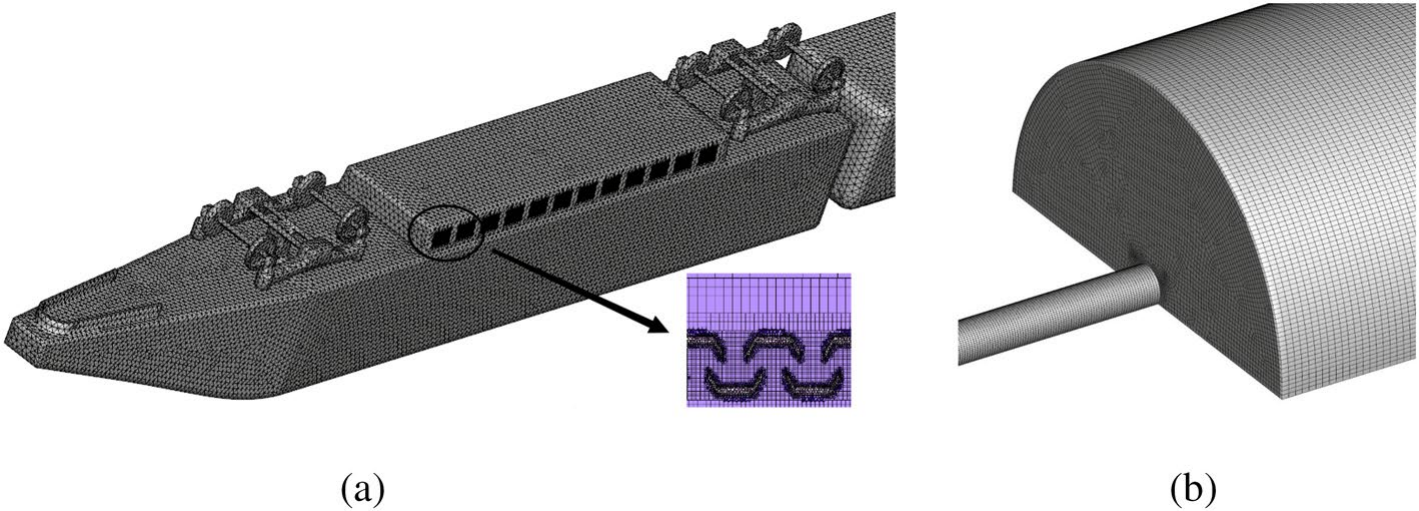
Train meshing strategy and calculation domain meshing strategy map.

To ensure the accuracy of the calculation and improve the accuracy of the analysis results, structured mesh technology is used to divide each area. In addition, due to the high wind and sand flow rate, small particle size, a wide range of concentration changes, and the need for sufficient mesh accuracy to characterize the effect of particles on grid and heat-generating equipment, double refinement method is used to refine the mesh in the train bypass area inside the tunnel. Furthermore, since the structure of the bogie, equipment cabin, and car connection is complex but has a significant impact on the flow field in the equipment cabin area, the grid of the car bottom structure is refined during mesh partitioning, and the grid at the equipment cabin grating is doubly refined. The grating V-V structure solid surface is set as a no-slip boundary condition, and the two sides of the calculation domain are set as wall boundary conditions, while the discrete phase boundary condition is set as a rebound boundary condition for the grating V-V structure. Finally, a flow calculation mesh with a total of 3.57 × 10^7^ units is obtained, and the mesh partitioning is shown in Fig. 5.

### Solution algorithm

#### Wind-sand flow movement model

In wind-sand flow, the wind speed profile is generally described using the Prandtl-Kármán logarithmic law distribution rate:

1 $$u_{y} = \frac{{u^{\prime}}}{k}\ln \frac{y}{{y_{0} }}$$

In the equation, *u*_*y*_ represents the wind speed at height *y* in m/s; *u'* represents the friction velocity in m/s; *k* is the Karman constant, which is typically taken as 0.4; *y*_*0*_ represents the aerodynamic roughness of the sand bed surface in meters, which can be calculated based on the Berggren 1/30 law^30^ and can be taken as d/30, where d is the sand grain diameter.

The movement of wind and sand generally takes three forms: suspension, saltation, and creep. The eddy dissipation coefficient in the wind and sand flow varies linearly with height, and the Euler equation describes the volume fraction distribution in the suspension layer as a function of the curtain, as shown in Eq. (2):

2 $$C(z) = C(z_{t} ) \cdot z^{ - b}$$

Unlike the layered movement of sand and dust when trains travel on an exposed track, the sand particles in a tunnel are highly irregular and discrete, and are constrained by the tunnel lining. Wind and sand movement inside a tunnel is irregular and hard to predict. Therefore, mathematical models such as those shown in Eqs. (1) and (2) are difficult to use to determine the specific movement patterns of the sand and dust.

#### Basic control equation of flow and Turbulence model

This study uses a 3D-DDES model for the HWS-LT scenario. This model was originally developed for the analysis of particle diffusion and had not been previously applied to train flow and heat transfer analysis. However, it is considered to have better performance in simulating transient wind and sand flows and is more suitable for problems involving the discrete movement of particles. It is an efficient and accurate numerical method^22,23^. The turbulent boundary layer is modeled using the unsteady RANS model with the following expression:

3 $$\frac{\partial \rho }{{\partial t}} + \frac{{\partial (\rho u_{i} )}}{{\partial x_{i} }} = 0$$

4 $$\frac{{\partial (\rho u_{i} )}}{\partial t} + \frac{{\partial (\rho u_{i} u_{j} )}}{{\partial x_{j} }} = \frac{\partial p}{{\partial x_{i} }} + \frac{\partial }{{\partial x_{j} }}\left[ {u(\frac{{\partial u_{i} }}{{\partial x_{j} }} + \frac{{\partial u_{j} }}{{\partial x_{i} }} - \frac{3}{2}\delta_{ij} \frac{{\partial u_{i} }}{{\partial x_{i} }})} \right] + \frac{\partial }{{\partial x_{j} }}\left( { - \rho \overline{{u^{\prime}_{i} u^{\prime}_{j} }} } \right) + \rho F$$

In the equation, *u* represents the velocity field, and *ρ*, *t*, *p* and *μ* represent Air density, Time, Pressure and Dynamic viscosity, respectively; *F* represents the force of particles under bidirectional coupling. The Navier–Stokes equations are filtered over time to obtain the LES control equations:

5 $$\frac{\partial \rho }{{\partial t}} + \frac{{\partial (\rho \tilde{u}_{i} )}}{{\partial x_{i} }} = 0$$

6 $$\frac{{\partial (\rho \tilde{u}_{i} )}}{\partial t} + \frac{{\partial (\rho \tilde{u}_{i} \tilde{u}_{j} )}}{{\partial x_{j} }} = \frac{{\partial \sigma_{ij} }}{{\partial x_{j} }} - \frac{{\partial \tilde{p}}}{{\partial x_{i} }} - \frac{{\partial \tau_{ij} }}{{\partial x_{j} }} + \rho F$$

In the equation,$\tilde{u}$ and $\tilde{p}$ represent the filtered velocity and pressure, respectively;$\sigma_{ij}$ represents the stress tensor caused by molecular viscosity;$\tau_{ij}$ represents subgrid-scale stress. The *k-ε* model is modeled using 3D-DDES^31^, which uses the following equation to calculate turbulence intensity:

7 $$\frac{\partial (\rho k)}{{\partial t}} + \frac{{\partial (\rho ku_{j} )}}{{\partial x_{j} }} = \frac{\partial }{\partial t}\left[ {(\mu + \frac{{\mu_{t} }}{{\sigma_{k} }})\frac{\partial k}{{\partial x_{j} }}} \right] + G_{k} + G_{b} - Y_{k}$$

8 $$\frac{\partial (\rho \varepsilon )}{{\partial t}} + \frac{{\partial (\rho \varepsilon u_{j} )}}{{\partial x_{j} }} = \frac{\partial }{{\partial x_{j} }}\left[ {(\mu + \frac{{\mu_{t} }}{{\sigma_{\varepsilon } }})\frac{\partial \varepsilon }{{\partial x_{j} }}} \right] + \rho C_{1} S \cdot \varepsilon - \rho C_{2} \frac{{\varepsilon^{2} }}{{k + \sqrt {v_{t} \varepsilon } }} + C_{1} \frac{\varepsilon }{k}C_{3} G_{b}$$

In the equation, *k* represents Turbulence Kinetic Energy, *ε* represents Turbulent Dissipation, *μ*_*t*_ represents Turbulent Viscosity, *v*_*t*_ represents Kinematic Viscosity,$\sigma_{k}$ and $\sigma_{\varepsilon }$ are the turbulent Prandtl numbers for *k* and *ε*, respectively, with $\sigma_{k}$ and $\sigma_{\varepsilon }$ taken as 1.0 and 1.2, respectively. *G*_*k*_ and *G*_*b*_ represent turbulence due to mean velocity gradients and buoyancy effects, respectively, where $G_{k} = u_{t} S^{2}$ and $G_{b} = 0$ in the expression. C_1_, C_2_ and C_3_ are constants with different numerical values, and more detailed explanations of their values can be found in the ANSYS help documentation^31^. In addition, for the DES model, the dissipation term is expressed as $Y_{k} = \frac{{\rho k^{3/2} }}{{l_{DES} }}$ and $l_{DES} = {\text{min}}(l_{rke} ,l_{LES} )$, where $l_{rke} = \frac{{k^{{_{3/2} }} }}{\varepsilon }$ and $l_{DES} = C_{des} \Delta_{\max }$, and *C*_*des*_ represents the model constant, which is taken as 0.61 in this case.$\Delta_{\max }$ represents the local maximum mesh spacing. To maintain the RANS model throughout the entire boundary layer, a 3D-DDES model is provided:

9 $$l_{DES} = l_{rke} - f_{d} \max (0,\,\,l_{rke} - C_{des} \Delta_{\max } \,$$

In the equation, *f*_*d*_ represents the Delay-function.

The energy conservation law here is actually the first law of thermodynamics. The energy *E* of a fluid in motion is the sum of its internal energy *i*, Kinetic energy $k = \left( {u^{2} + v^{2} + w^{2} } \right)$, and potential energy *P*. The energy conservation equation in terms of temperature is:

10 $$\frac{{\partial \left( {\rho T} \right)}}{\partial t} + {\text{div}}\left( {\rho uT} \right) = {\text{div}}\left[ {\frac{\lambda }{{C_{p} }}{\text{grad}}T} \right] + s_{T}$$

In the equation, *C*_*p*_ represents the specific heat capacity, *T* represents temperature, *λ* represents the thermal conductivity, and *S*_*T*_ represents a customized volumetric heat source that includes the dissipative term due to viscosity.

#### DPM and heat transfer model

The wind-sand movement considered in this study consists of three stages: "wind-sand jumping", "sand particles suspension", and "sand impacts on vehicle or tunnel walls, and going in and out of equipment cabin." Due to the effects of the enclosed tunnel environment and the flow field surrounding the train, the wind-sand movement at this time is irregular compared to the stable flow mode in open environments, and in any stage, the sand particles are discrete rather than continuous phases. Therefore, the sparse discrete phase model in Euler–Lagrange coordinate system is used in this study to describe and track the movement of sand particles in the flow field. The DPM model generally requires that the volume fraction of the particle phase is less than 10%, and the range of sand concentrations in this study meets this standard. The momentum equation for sand particles is expressed as:

11 $$\frac{{du_{p} }}{dt} = F_{D} \left( {u_{a} - u_{p} } \right) + \frac{{g(\rho_{p} - \rho_{a} )}}{{\rho_{p} }} + F_{a}$$

In the equation, *u*_*p*_ and *u*_*a*_ represent the velocities of the sand particles and air, respectively.$\rho_{p}$ and $\rho_{a}$ represent the density of the sand particles and air, respectively. *F*_*D*_ represents the drag coefficient, *F*_*a*_ represents the additional force considered in numerical simulations, and *g* is taken as 9.81 m/s^2^.

The gravitational force acting on sand particles in this study follows the law of universal gravitation, while lift force follows Saffman's law, and drag force follows Stokes' law^31^. The interphase momentum and heat exchange are computed using the Sub-relaxation scheme model. This study provides an interphase coupling calculation model, where the particle momentum change is given by:

12 $$F = \sum {\left[ {\frac{{18\beta \mu C_{D} {\text{Re}}}}{{\rho_{p} d_{p}^{2} 24}}\left( {u_{p} - u_{a} } \right) + F_{other} } \right]} \mathop {m_{p} \Delta t}\limits_{{\phantom{0}}}^{ \bullet }$$

In the equation,$\rho_{p}$ denotes the particle density.$D$ denotes the particle diameter. Re denotes the relative Reynolds number. *C*_*D*_ represents the drag coefficient.$\mathop {m_{p} }\limits^{ \bullet }$ represents the particle mass flow rate.$\Delta t$ represents the time step. *F*_*other*_ denotes other interphase forces. This momentum exchange will act as a 'summation' of momentum applied in the subsequent calculation of fluid phase momentum balance.

Turbulent dispersion of particles can be modeled by the Discrete Random Walk (DRW) model:

13 $$u{^{\prime}}_{p} = \zeta \sqrt {u{^{\prime}}_{a}^{2} } = \zeta \sqrt {2k/3}$$

where *ξ* is the normally distributed random number^32^.

In the case of *S*_*T*_ (Eq. 10), the energy source terms caused by radiation and interphase energy transfer are described as follows:

In this study, the DPM particle type is determined to be inert, assuming that the initial temperature of the particles, denoted as *T*_*l*_, satisfies the condition *T*_*l*_ < *T*_*vap*_ , which represents the theoretically calculated particle volatilization temperature denoted as *T*_*vap*_. Sand particles are considered to not participate in any reaction processes and have no mass exchange with the gas phase. Therefore, the processes of deposition, boiling, or evaporation of DPM are not considered in this study in order to strictly satisfy the condition *T*_*l*_ < *T*_*vap*_ in the computational process. The value of *T*_*vap*_ is set as a theoretically feasible maximum value. Under the aforementioned assumptions, we assume that the temperature inside and outside the particle is consistent. Therefore, a simple heat balance equation is adopted to relate the particle temperature function *T*_*p*_(*t*) with the heat transfer phenomena at the particle surface:

14 $$m_{p} c_{l} \frac{{dT_{p} }}{dt} = h_{g} \pi D^{2} (T_{k} - T_{l} ) + \varepsilon_{p} A_{p} \sigma (\theta_{R}^{4} - T_{l}^{4} )$$

In the equation, *m*_*p*_, mass of the particle, kg; *c*_*l*_, heat capacity of the particle, J/(kg·℃); *T*_*k*_, local temperature of the continuous phase, K; *ε*_*p*_, particle emissivity; *h*_*g*_, convective heat transfer continuous phase, W/m^2^·K; *σ*, Stefan-Boltzmann constant, 5.67 × 10^8^ W/m^2^·K; $\theta_{s}$, radiation temperature, $\left( {\frac{G}{4\sigma }} \right)^{1/4}$(K); *G* is the incident radiation in W/m^2^:

15 $$G = \smallint_{\Omega = 4\pi } Id\Omega$$

In the equation, *I* is ten radiation intensity and *Ω* is the solid angle. *h*_*g*_ is evaluated using the correlation of Ranz and Marshall^33,34^:

16 $$h_{g} = \frac{{2 + 0.6{\text{Re}}_{D}^{1/2} \Pr^{1/3} }}{{k_{\infty } /D}}$$

In the equation, $k_{\infty }$, thermal conductivity of the continuous phase, W/(m·K); Re_d_, Reynolds number based on the particle diameter and the ralative velocity; Pr, Prandtl number of the continuous phsae.

Assuming that the particle temperature changes approximately linearly over a continuous integration time, integrating Eq. (10) with respect to time, we obtain:

17 $$m_{p} \frac{{dT_{p} }}{dt} = A_{p} \{ - [h + \varepsilon_{p} \sigma T_{p}^{3} ]T_{p} + [hT_{\infty } + \varepsilon_{p} \sigma \theta_{R}^{4}$$

By using Fluent, iterate integration for Eq. (17) with a time step represented by $\Delta t$ to obtain the particle temperature at the next moment:

18 $$T_{1} (t + \Delta t) = [T_{1} (t) - \alpha_{p} ]e^{{ - \beta_{p} \Delta t}}$$

19 $$\alpha_{p} = \frac{{h_{g} T_{\infty } + \varepsilon_{p} \sigma \theta_{R}^{4} }}{{h_{g} + \varepsilon_{p} \sigma T_{1}^{3} (t)}}$$

20 $$\beta_{p} = \frac{{A_{p} (h_{g} + \varepsilon_{p} \sigma T_{1}^{3} (t))}}{{m_{p} c_{1} }}$$

According to Eqs. (10) to (20), when sand particles pass through the computational grid, the heat absorbed (or released) by the sand particles is treated as a source term in the governing energy equation of the continuous phase, achieving heat transfer between solid particles and the gas phase. In this study, component diffusion and chemical reaction-related heat source variations are not considered, and a custom model is used to implement heat transfer induced by interactions between solid phase and solid particles. For thermal conduction solution, Fluent utilizes a simplified thermal conduction integration equation built-in within the software. For solid media with properties of absorption, emission, and scattering, the radiative heat transfer equation (RTE) along direction $\overrightarrow {s}$ at position $\overrightarrow {r}$ is applied.

21 $$\frac{{dI\left( {\overrightarrow {r} ,\overrightarrow {s} } \right)}}{ds} + \left( {\alpha + \sigma_{s} } \right)I\left( {\overrightarrow {r} ,\overrightarrow {s} } \right) = \alpha n^{2} \frac{{\sigma T_{k}^{4} }}{\pi } + \frac{{\sigma_{s} }}{4\pi }\int_{0}^{4\pi } {I\left( {\overrightarrow {r} ,\overrightarrow {{s^{\prime}}} } \right)} \Phi \left( {\overrightarrow {s} ,\overrightarrow {{s^{\prime}}} } \right)d\Omega^{\prime}$$

In the equation, $\overrightarrow {r}$, $\overrightarrow {s}$, and $\overrightarrow {{s^{\prime}}}$ represent position vector, direction vector, and heat dissipation vector, respectively. *s* represents the path length, *α* and *n* represent absorption coefficient and refractive coefficient, respectively, and $\sigma_{s}$ represents scattering coefficient. $I\left( {\vec{r},\vec{s}} \right)$ is a radiative intensity function that depends on $\overrightarrow {r}$ and $\overrightarrow {s}$, and $\Phi (\vec{s},\vec{s}^{\prime})$ is a phase function that also depends on $\overrightarrow {s}$ and $\overrightarrow {{s^{\prime}}}$. When *α* is used as an input, the total heat source induced by radiation can be expressed as a sum:

22 $$- \nabla \mathop q\nolimits_{r} = aG - 4a\sigma \mathop {T_{s} }\nolimits^{4}$$

*qr* represents the radiative heat flux, and this expression can be directly substituted into Eq. (10). For particles with properties of absorption, emission, and scattering, the transport equation for incident radiation is expressed as:

23 $$\nabla \cdot \left[ {\frac{\nabla G}{{3\left( {\alpha + \sigma_{s} } \right) - C\sigma_{s} }}} \right] + 4\pi \left( {a\frac{{\sigma \mathop {T_{s} }\nolimits^{4} }}{\pi } + \mathop E\nolimits_{p} } \right) - (a + \mathop a\nolimits_{p} )G = 0$$

Here, *Ep* represents the equivalent radiation of the particle, *αp* represents the equivalent absorption coefficient of the particle, and *C* is a coefficient for the linear anisotropic phase function, all of which are defined in the Fluent documentation. Therefore, in the *ST* term of Eq. (10), the expression for the heat source term caused by particle radiation is given by:

24 $$- \nabla q_{r} = - 4\pi \left( {a\frac{{\sigma T_{s}^{4} }}{\pi } + E_{p} } \right) + \left( {a + a_{p} } \right)G$$

This study assumes that the heat source wall is a diffuse gray surface, and the wall emissivity is denoted $\rho_{w}$ as 1-*εw*. The incident radiative heat flux *G* on the wall is then represented as:

25 $$- q_{r,w} = \frac{{\varepsilon_{w} }}{{2\left( {2 - \varepsilon_{w} } \right)}}\left( {4\sigma T_{w}^{4} - G_{w} } \right)$$

### Parameter setting

The maximum speed of HST specified in the Lanxin railway is considered as the operational speed of HST in this study. With a fixed wall surface as the reference frame, *V* represents the operating speed of the HST, which is 250 km/h. The natural wind speed follows the logarithmic distribution of Karman, with an average velocity of 15 m/s. The surface roughness of the equipment is represented by *ra*, which is 0.5. The roughness height of the equipment compartment, ha, is set at 0.001 m. The specific heat capacity of the sand particles is taken as 1.22 × 103 J/(kg·℃). *Tk* and *Tl* are taken as 309.15 K. We represent the sand particle concentration in the calculation domain with the volume fraction of sand, denoted as *φ*, which is 0 (condition 1), 0.0001 (condition 2), 0.001 (condition 3), and 0.01 (condition 4). Table 1 presents the distribution of different particle sizes *D* on the surface under three average wind speed conditions during windy and sandy weather. The particle size distribution of sand follows a normal distribution, with predominantly medium and fine sand. Additionally, due to the constraint of the threshold wind speed, sand particles larger than 0.5 mm are not considered in this study^35^.

**Table 1:** Particle size distribution range.

*D* (mm)		0–0.05	0.05–0.08	0.08–0.20	0.20–0.50	> 0.50
Percentage	8.0 m/s-10.0 m/s	3.02e-01%	6.12e + 00%	1.43e + 01%	2.49e + 01%	*
	18.9 m/s-21.5 m/s	8.96e-02%	7.34e + 00%	5.41e + 01%	3.25e + 01%	
	31.2 m/s-36.2 m/s	7.23e-01%	1.21e + 01%	6.54e + 01%	5.11e + 01%	

## Verification of results

**Figure 6:**
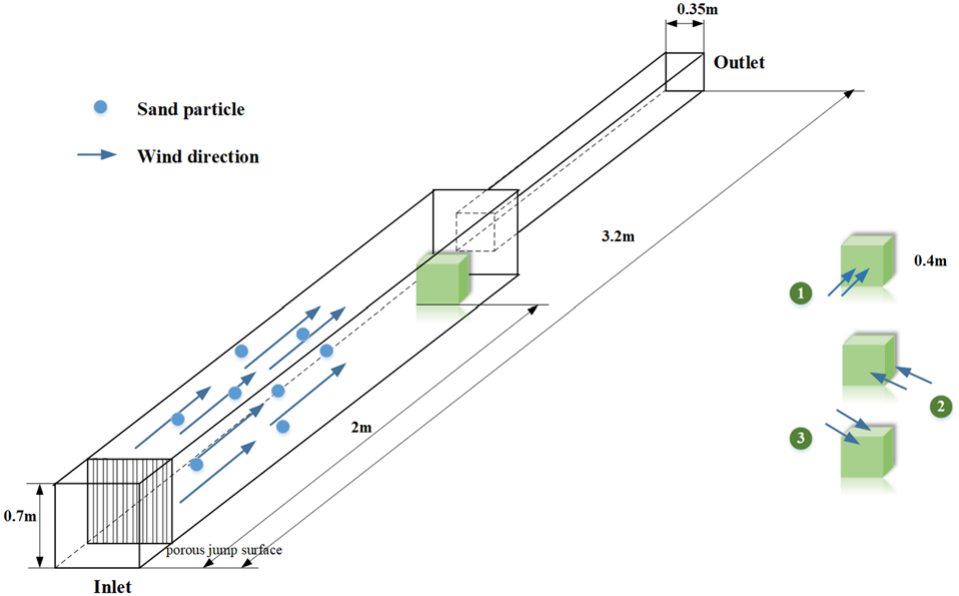
Boundary conditions for the HWS-EB scenarios.

It is necessary to explore the mechanism of the impact of wind-sand flow on the heat dissipation of individual equipment in the equipment cabin and to validate the feasibility of numerical calculations in the HWS-LT scenario. Similar to the studies conducted by Jia et al.^20^, Niu et al.^28^, Song, and Mikhailenko et al.^36,37^ in the field of fluid-thermal coupling, this study established a "hybrid-phase impact equipment block" scenario (referred to as the "HWS-EB" scenario) as shown in Fig. 6.

The cubic block represents the heat source in the equipment chamber, with a predefined heat flux density of 100 W/m^2^. The front end of the external field is set as the inlet and release surface of discrete phase, while the back end is the outlet, and the remaining surfaces are walls. The specific parameters in the fluid domain are shown in Fig. 6.

To ensure that the model is simplified within a reasonable range, the scenario in this study has the following characteristics:

- In order to replicate the rectifying effect of the grille and filter screen, and correspond with the grille network in the previous HWS-LT scenario, a porous jump surface, which can model a thin membrane with known velocity/pressure-drop characteristics, is adopted to simplify the modeling of the grilles^20,38^.
- Considering the impact of irregular vortex structures on the heat dissipation of the equipment, the P1 and P2 cubic fluid domains are connected end to end, and the equipment block is placed 0.2 m upstream of the P2 fluid domain, where wind-blown sand can form vortex flow accumulation downstream of the P1 fluid domain, allowing the wind-blown sand to fully surround the equipment block. The settings of P1 and P2 can effectively replicate the fluid environment of the equipment block in the equipment cabin, especially for the auxiliary converter located near the corners of the equipment cabin.
- In the HWS-LT scenario, the pressure distribution inside the equipment cabin is not uniform. In such cases, the projection of the heat-generating equipment on the windward side is influenced by the flow direction, resulting in various parameter uncertainties. Therefore, in order to maximize the likelihood of equivalence, based on the principle of similarity, the flow direction is changed with respect to the equipment block as the reference frame to achieve correspondence between the two scenarios in terms of flow characteristics, Fig. 6 on the right shows the variation in inflow direction.
- The LES model with low Reynolds number is used for turbulence modeling, while the DPM is used for the particle phase. The gas phase is set as compressible steady-state flow. The mesh around the equipment block is refined, with the total number of mesh elements being 9.73 × 10^5^. Table 1 provides a detailed description of the specific parameters.
- The range of the average particle diameter varies within the statistical range of the data in Table 2. The values of surface roughness are determined according to the standard GB/T 17,851–2022^39^. The parameters for air temperature, Particle temperature, and Particle specific heat capacity are shown in Table 2.

**Table 2:** Parameter settings in the HWS-EB scenarios.

Parameter	Describe parameters
Water intake flow velocity *v*_*eb*_/km/h	1, 2, 3, … 11, 12
Incidence angle *α*_*ed*_	Direction 1, Direction 2, Direction 3
Roughness height *h*/ m	0.0005, 0.001, 0.002
DPM concentration *φ’*	0.01, 0.005, 0.001
Granular diameter *D*/ mm	0.001, 0.1, 0.3

## Analysis and results

To clarify the effect of heat accumulation in the HST equipment chamber under the HWS-LT scenario and better analyze the influencing mechanism of the heat dissipation of equipment under the effects of wind-sand flow and no-sand wind in the tunnel space, a systematic analysis of the characteristics of millimeter-scale wind-blown sand flow, particle dispersion, concentration changes in the chamber under transient airflow effects, and potential evolution mechanisms should be conducted before analyzing the temperature field in the chamber under multi-field coupling. The research by Hatami shows that the influence of fluid flow characteristics on heat transfer is more significant^40^.

### Flow field

In actual engineering scenarios, wind-blown sand is released from a location far from the tunnel entrance and gradually enters the tunnel space under the dual effects of natural wind and gravity. To simulate the flow characteristics of wind-blown sand entering the tunnel space in actual engineering scenarios as accurately as possible and further clarify the sand movement in the tunnel, the external flow field under steady state mode is first analyzed, followed by the analysis of the internal and external flow field under unsteady state mode.

### Steady-state—external flow field

**Figure 7:**
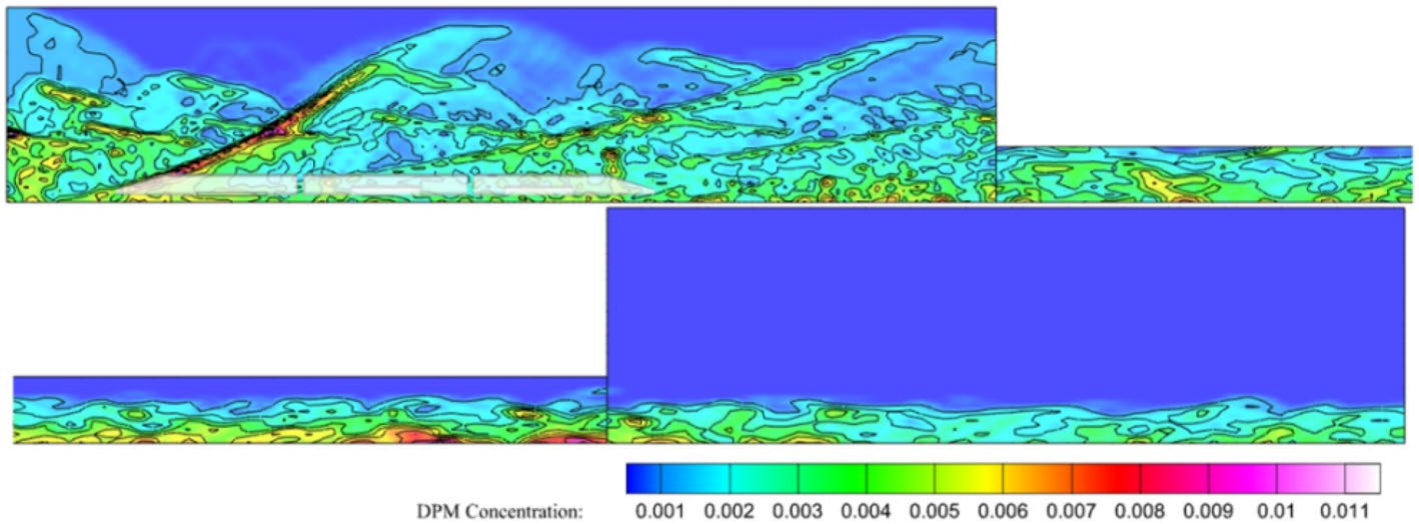
Concentration distribution cloud map.

Figure 7 shows the diffusion map of wind-sand flow after entering the tunnel from an open environment in the steady state mode. Wind-blown sand forms a relatively stable state after entering the tunnel. Inside the tunnel, the wind speed is relatively low due to the obstruction of the tunnel walls, making the movement of sand particles relatively stable. Sand particles move in different forms, including saltation, suspension, creep, and so on^41^. The existence of small vortex structures inside the tunnel leads to an increase in the concentration of sand particles along the longitudinal range of the tunnel, making it easier for sand particles to accumulate on the track. Over time, sand particles will gradually accumulate, forming a phenomenon called sand accumulation. For the equipment chamber, wind-blown sand with creeping and jumping movements that have vortex structures is more likely to enter the equipment chamber. Therefore, when HST travels in the long tunnel space of high wind-blown sand areas, the influence of wind-blown sand must be considered.

#### Non-steady-state—external flow field

**Figure 8:**
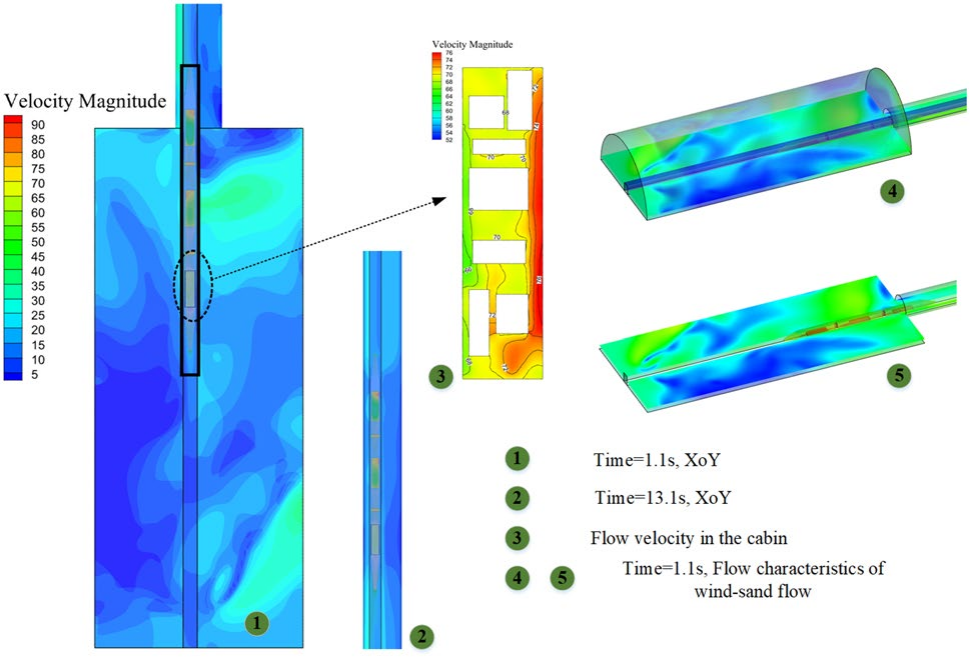
Cloud diagram of the external flow field of the train at 1.1 s and 13 s.

In the HWS-LT scenario, the train head carriage enters the tunnel at 0.72 s, where the high-speed flow around the train body disturbs the wind and sand that gathers at the tunnel entrance. This causes a high concentration of wind and sand to strike the equipment compartment, with some sand particles entering the compartment through the grid and filter net. The HST equipment compartment carries sand into the tunnel with an initial concentration of *φ*_0_ > 0, as shown in Fig. 8.

**Figure 9:**
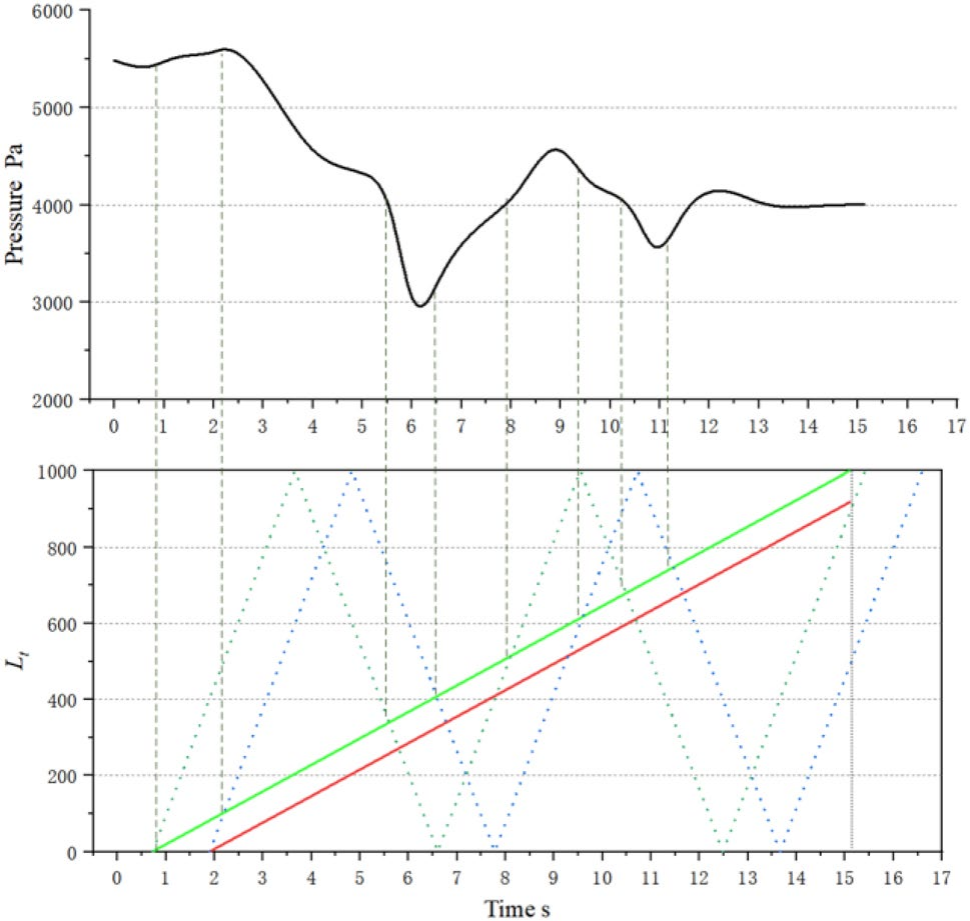
Pressure and tunnel pressure wave change process.

The air in front of the train is severely compressed, causing the pressure at measuring points to rapidly increase and form a compression wave at the tunnel entrance. The compression wave travels at the speed of sound towards the exit of the tunnel, carrying some sand particles with it and reducing the concentration of sand. At 1.89 s, the train tail enters the tunnel and similarly generates an expansion wave at the tunnel entrance, which also travels at the speed of sound towards the exit of the tunnel, sweeping along some sand particles. The two waves then begin to propagate and reflect inside the tunnel, intersecting and colliding with the train multiple times, causing the pressure on the train body and equipment compartment to constantly change. Additionally, the pressure on the train surface periodically decreases, rises, and then drops with an unpredictable cycle, as the diffusion of the flow field around the train is restricted by the tunnel wall, and the intrusion of wind-blown sand can occur with varying pressures. The average pressure is nearly 2 × 10^3^ Pa lower than when traveling on a plain track. This phenomenon is particularly prominent when the train is traveling through long or super-long tunnels, as the pressure drop is more pronounced, leading to poor ventilation and heat dissipation, and resulting in the accumulation of a large amount of thermal energy in the train's heat-generating equipment in a short period of time. The pressure at measuring points is shown in Fig. 9.

**Figure 10:**
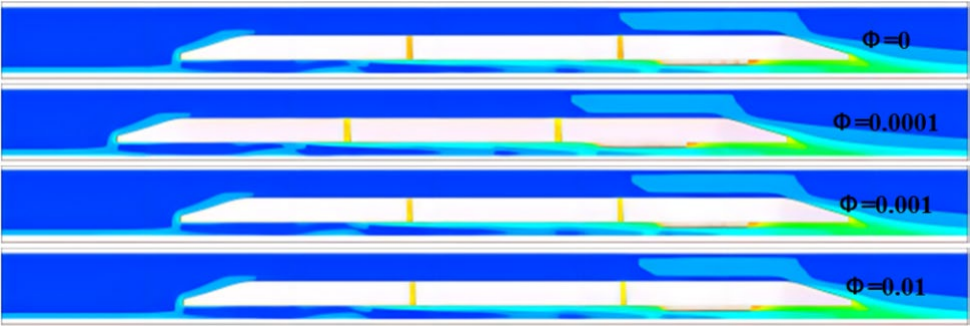
Flow around train.

Figure 10 shows the external flow field of the train under four concentration gradients (Discrete phase initial concentration). The green solid line represents the position (longitudinal) of the train's leading nose tip at each moment. The red solid line represents the position of the train's trailing nose tip at each moment. The green dashed line represents the position (longitudinal) at which the compression wave generated by the leading car propagates to at each moment. The red dashed line represents the position at which the expansion wave generated by the trailing car propagates to at each moment. The equipment compartment is installed in the tail carriage of the train, and the display is located at a horizontal section *X* = 3.1 m after the train has traveled for 5 s(292.3 m). The airflow separates from the train's nose, while the sand particles move with the airflow and separate near the nose. The strong sand-carrying airflow generated around the train gradually expands along the direction of the train body, and gradually begins to dissipate and weaken in the wake region, while the flow velocity changes more drastically at the windscreen of the equipment compartment, forming stagnation points.

Due to the raised edges of the equipment compartment, high-speed vortices accumulate downstream (towards the train's tail), with velocities higher than the flow velocity underneath the equipment compartment. The sand particles are affected by the vortex structure and exhibit a lifted state. It is worth noting that the higher the concentration *φ*, the lower the overall flow velocity of the train's external flow field. Due to the bidirectional coupling adopted in the simulation calculation, the influence of both continuous relative particles and the effect of particles on the continuous phase are taken into account. Therefore, due to the strong airflow generated around the train being dragged by the sand particles, the sand particles accelerate and gain more momentum, then leap up and suspend in a short period of time. The higher the sand particle concentration (the volume fraction of sand particles per unit volume), the greater the impact on the airflow, and vice versa.

**Figure 11:**
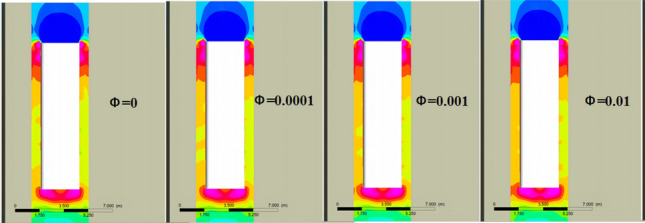
Pressure distribution of flow field outside the equipment cabin.

**Figure 12:**
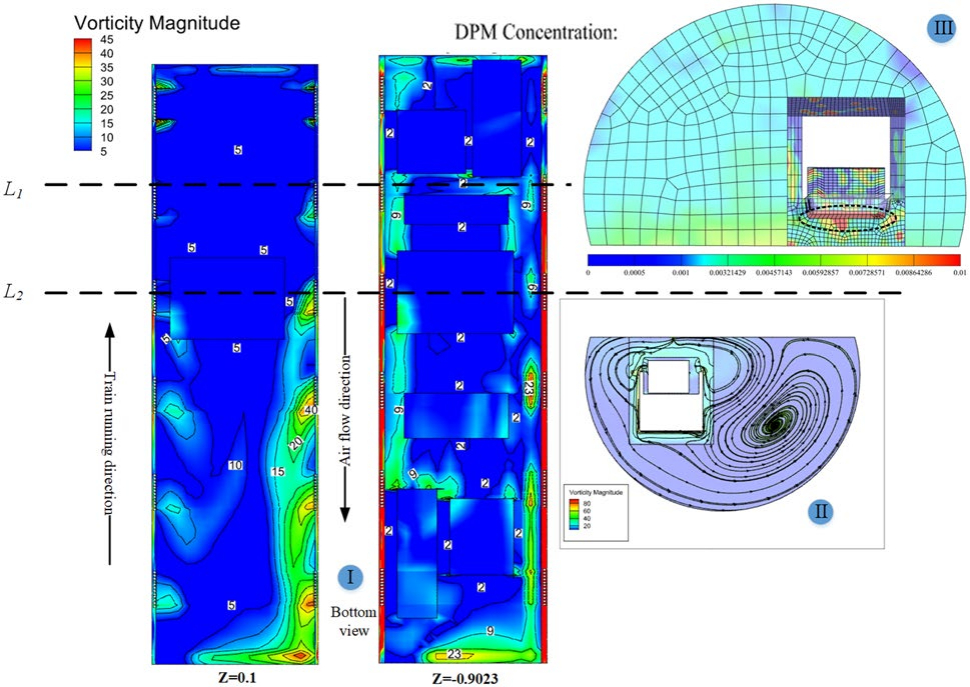
Internal and external flow field of equipment cabin.

In a single-bore double-track tunnel, HST usually travels on the right side, and the distance from the train to the tunnel wall on both sides (Far wall L, Near wall R) is different. The narrow environment on the R side leads to a smaller circulation area on that side, while the circulation area on the L side is larger. As shown in Fig. 11, the pressure on both sides of the equipment compartment is distributed asymmetrically. At the same time, noticeable vortices and vortex detachment structures are formed in the wake region of the train (As shown in II of Fig. 12), and the distribution of the vortex structures affects the pressure distribution on both sides of the train. During this period, the pressure on the R side of the equipment compartment experiences a sudden increase and oscillating change, causing significant fluctuations in pressure on both sides of the equipment compartment and showing a situation where the pressure on the L side is higher than the R side. It is worth noting that this pressure situation is relatively inconspicuous when *φ* = 0. However, as *φ* increases, the pressure on both sides gradually decreases, with the decreasing trend on the R side being more pronounced. This is because the open area on the far wall side is larger, and the sand particles are driven by the flow formed around the train, resulting in a lower local sand concentration on the R side than the L side at the same time. Under the restriction of the tunnel and train wall, the higher the concentration, the more obvious the asymmetric situation of sand accumulation on the outer side. The sand is accustomed to moving to the outside (As shown in III of Fig. 12), resulting in asymmetric volumes and flow velocities of windblown sand entering both sides of the equipment compartment. Figure 12 shows the asymmetric phenomenon of windblown sand invading the equipment compartment (*φ* = 0.001).

#### Non-steady-state—internal flow field

**Figure 13:**
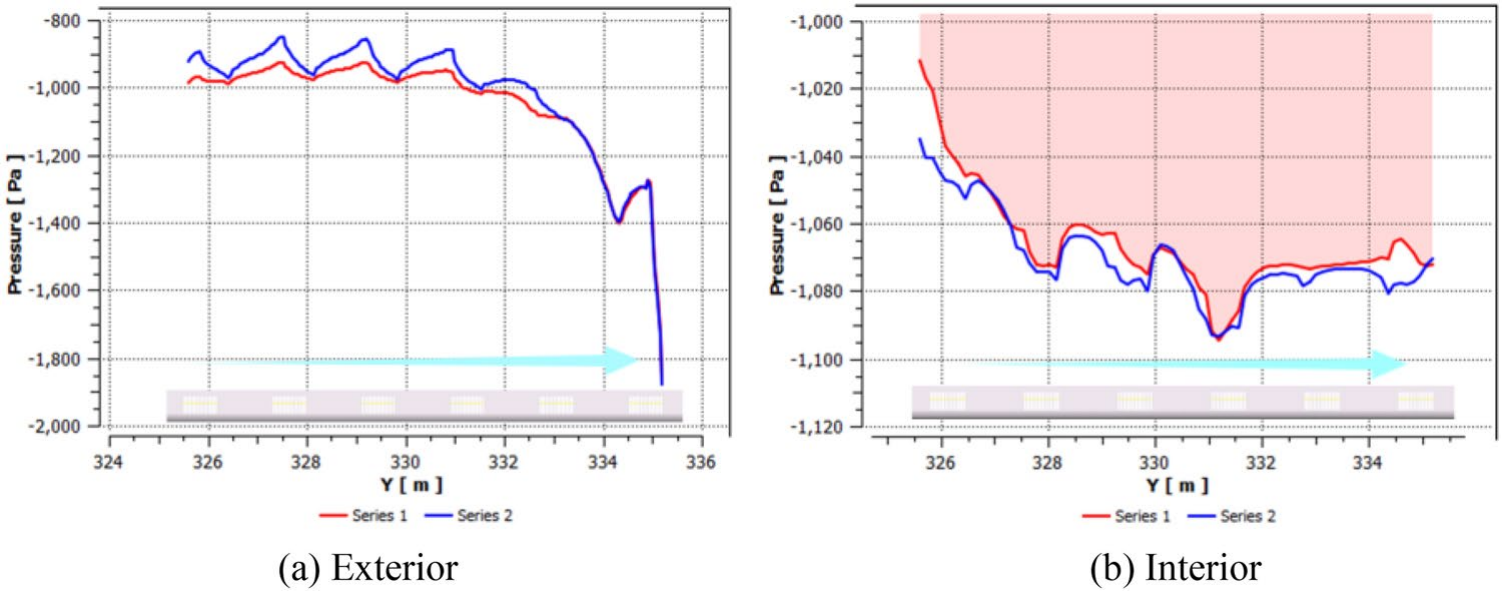
Pressure changes with position plot, (**a**) Exterior, (**b**) interior.

The windblown sand flow reaches the front panel of the equipment compartment after passing through the head car, middle car, and arriving at the tail car, where it is obstructed and the velocity drops sharply, forming a stagnant flow with high negative pressure, as shown in Fig. 13a. After the windblown sand flow passes around the front panel of the equipment compartment, it flows towards both sides of the equipment compartment, and the velocity gradually increases. The airflow entering the equipment compartment through the upstream grille has a lower velocity, but the airflow velocity outside the R side of the compartment increases, causing the airflow velocity entering the equipment compartment through the downstream grille to gradually increase. The driving conditions in the tunnel are not conducive to grille-air circulation, which differs greatly from the situation when the train is running on a clear track. This is confirmed by the flow field diagram (I) inside the equipment compartment shown in Fig. 12.

**Figure 14:**
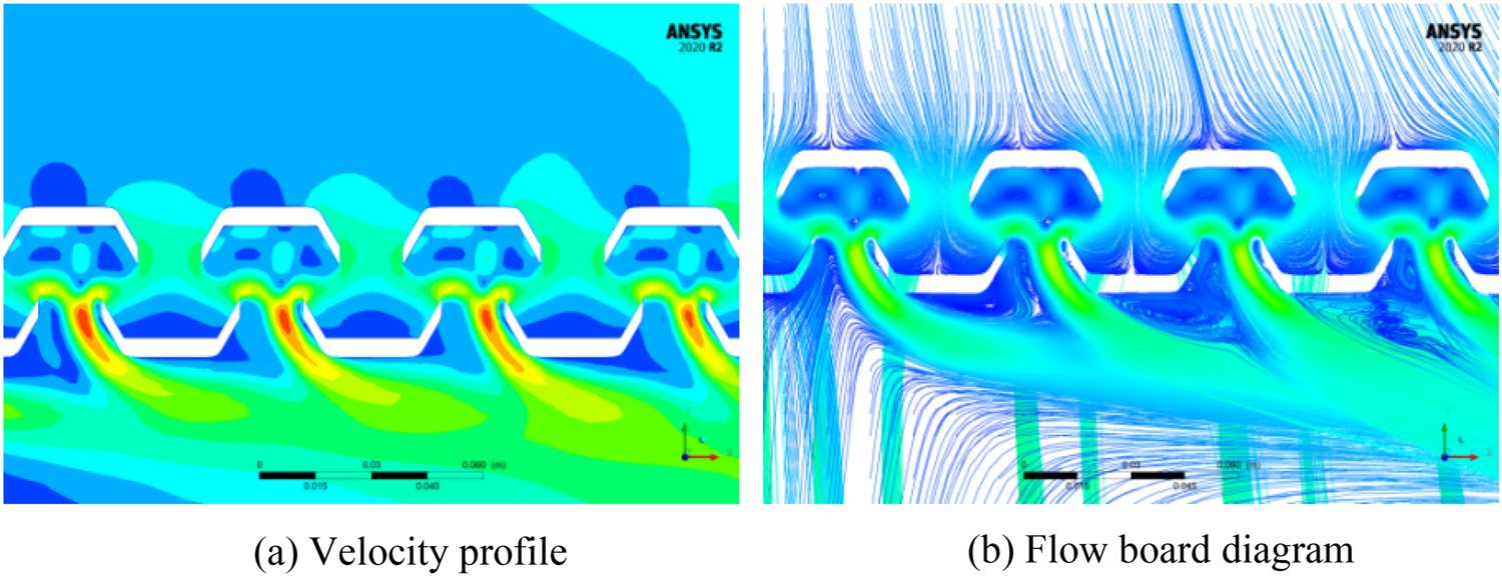
Rectification effect of grid, (**a**) Velocity profile, (**b**) Flow board diagram.

As shown in Fig. 13, the pressure on the L side is significantly higher than that on the R side in the outer pressure, while in the inner pressure, the opposite situation occurs. This results in a greater pressure difference between the inside and outside of the L side, and a more severe intrusion of windblown sand (coincidentally, the sand concentration near the ground is higher on the L side at this time). The pressure inside and outside of the upstream grille is significantly lower than that of the downstream grille, and a negative pressure is formed in front of the grille. This causes the air carrying sand particles to quickly flow downstream towards the equipment compartment after entering the grille, which in turn causes significant turbulence at the upstream "equipment-to-equipment" or "equipment-to-compartment wall" and more violent flow of windblown sand downstream. The above description can be clearly seen from Fig. 14. The V-V structure plays a transitional pressure role during this period, which results in a relatively low flow velocity entering the equipment compartment.

**Figure 15:**
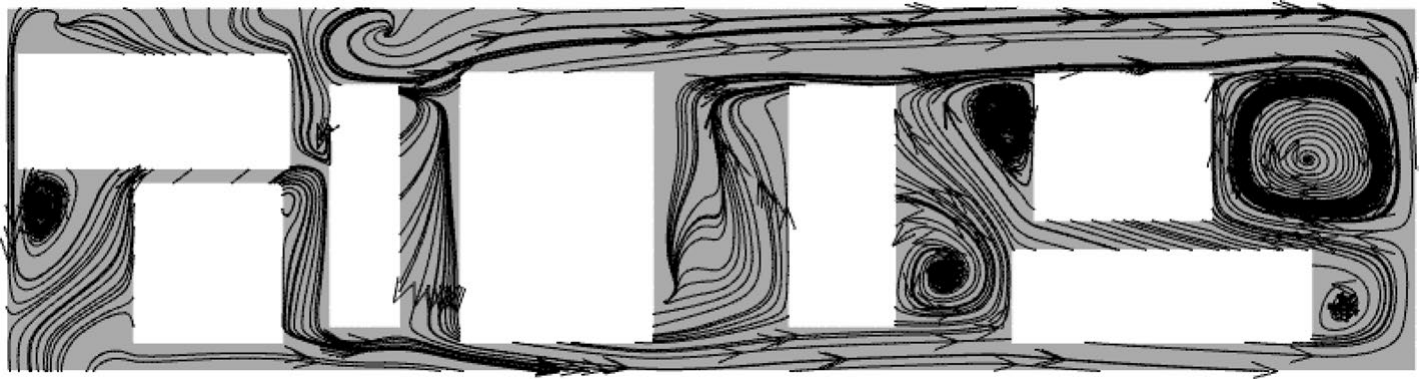
Wind-sand flow streamline diagram in the cabin, Z = -0.9023.

When HST is traveling in a tunnel, the internal pressure of the equipment cabin is also influenced by the tunnel pressure wave. However, pressure changes occur but are all below 0.1 kPa and do not have a significant impact. The relative velocity of gases inside the equipment compartment is less than 12 m/s. At this point, the windblown sand flow characteristics differ from natural wind characteristics that do not contain sand particles. Most of the natural wind directly sweeps along the side walls of the equipment compartment, without directly impacting the heat-generating equipment. However, the windblown sand flow contains heavier discrete matter, which, after passing through the grille with the gas phase, due to its larger mass and greater inertia, directly impacts some of the equipment, while the rest of the windblown sand flow forms large vortex structures at the closed right-angle structures on both sides of the equipment compartment, as shown in Fig. 15. The degree of concentration of discrete matter increases the intensity of these structures.

**Figure 16:**
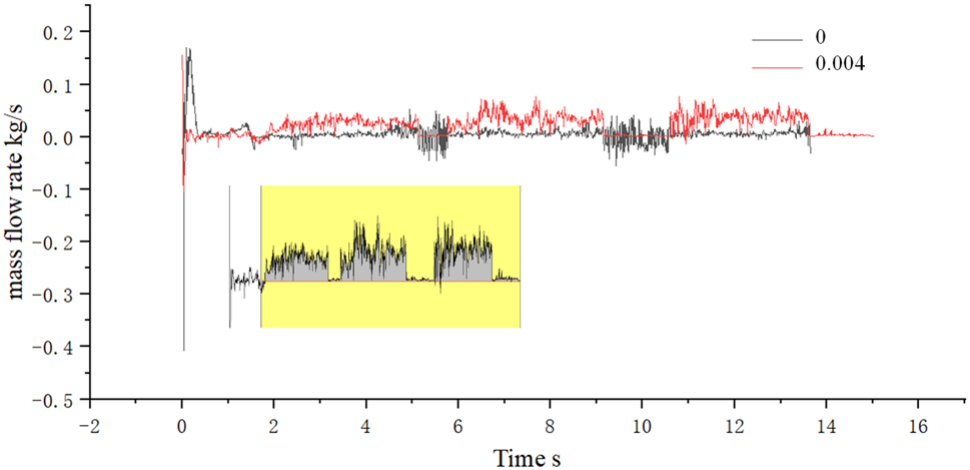
Mass flow.

We conducted mass flow rate monitoring for all grilles and sand leakage holes in the equipment compartment. The comparison of mass flow rates (*ρ*_*Q*_) for all grilles under two conditions, *φ* = 0 (no sand) and *φ* = 0.004 (windblown sand), is shown in Fig. 16. Under the condition *φ* = 0, the inflow and outflow *ρ*_*Q*_ are basically balanced (With a small variation in the range of 0 kg/s), and the amplitude of the change is small after reaching a steady state. When sand particles are present (*φ* = 0.004), the sand particles increase the particle concentration inside the equipment compartment, thereby increasing the fluid density. Thus, the amplitude of the *ρ*_*Q*_ curve in Fig. 16 is larger when *φ* = 0.004 (In other words, the inflow *ρ*_*Q*_ is relatively higher in a unit time). In addition, due to the larger mass of individual sand particles and their inertia, they tend to accumulate at blocking walls. Some sand particles form a blockage effect on grilles or filter screens, resulting in a decrease in the outflow *ρ*_*Q*_. Since the flow velocity inside the compartment is small, the sand particles gradually settle on the bottom surface of the equipment cabin under time-varying conditions (As demonstrated in Fig. 12, section III). It must be emphasized that the description of sand particle deposition in this paper practically refers to the state where the momentum of the sand particles decays to a sufficiently small value and they are unable to leap up again within a short period of time. This description is a way of capturing the practical engineering background. We evaluated the accumulation degree of sand particles using area integration. When HST departed from the "*W*_*t*_ = 1 km–*φ* = 0.004" scene, the total sand dust accumulation in the equipment cabin during the traveling process was *m*_*s*_ = 321.63 g.

**Figure 17:**
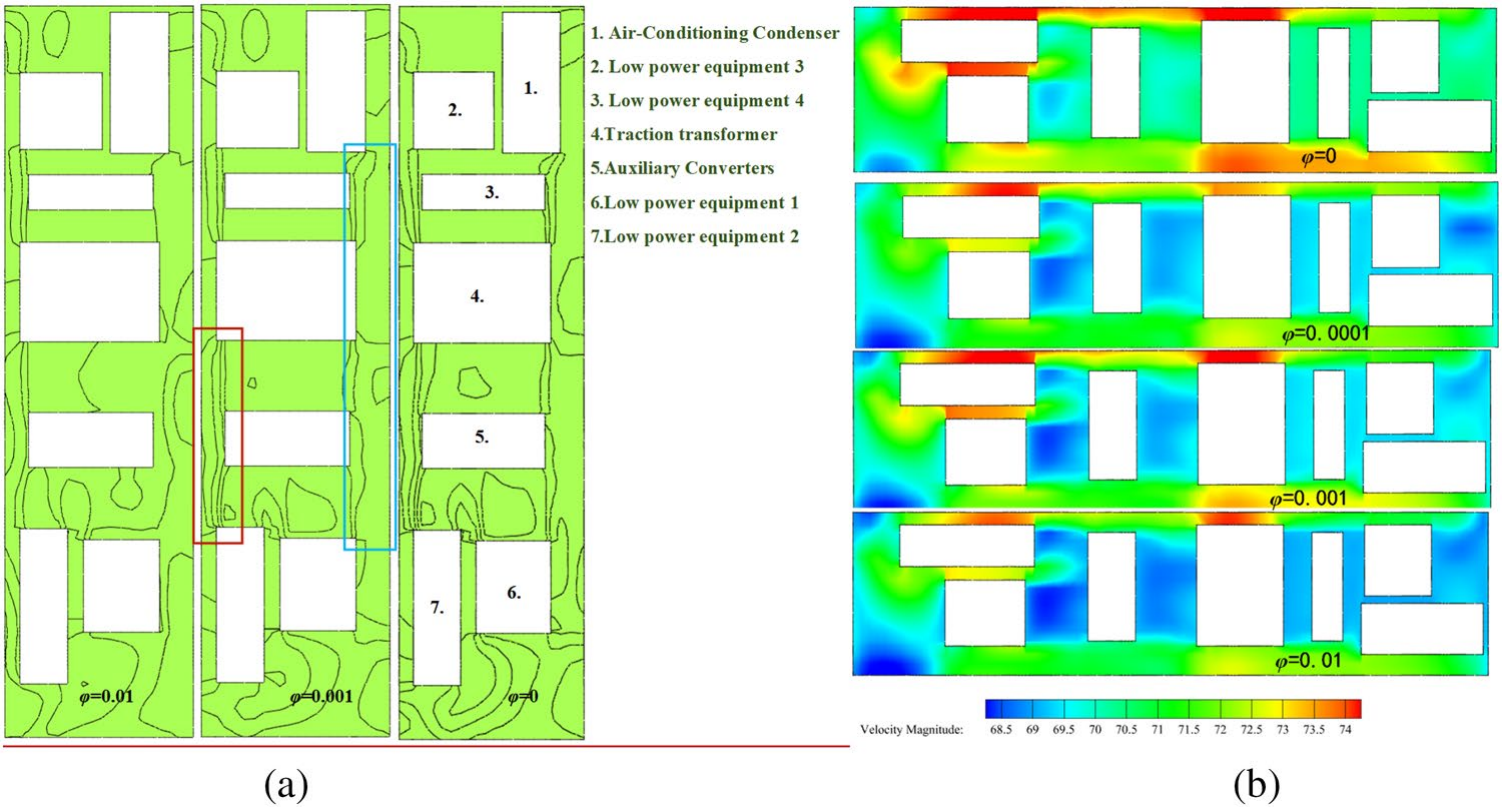
The contour map of flow velocity at Z = -0.9023; The velocity distribution chart at Z = -0.5 (using a fixed area as a reference system).

Figure 17 shows the flow characteristics under four concentrations. On the same contour map, the denser the distribution of contour lines, the faster the velocity changes (red box in Fig. 17a); the sparser the distribution of contour lines, the slower the velocity changes (blue box in Fig. 17a). The complicated flow area mainly surrounds the walls of the equipment compartment, the flow velocity at the grille is relatively high, and the flow velocity around the air conditioning condenser located at one end of the equipment compartment and around low-power equipment 3 and 4 is relatively low, forming some larger vortex structures and carrying sand particles, resulting in a higher concentration downstream of the equipment compartment than upstream. This is due to the restriction of sand particles caused by vortex structures. A portion of sand particles moves downstream of the equipment compartment following the wind vector direction, hovering again at each device assembly position for a period of time before flowing out of the equipment compartment through the downstream grille, taking away heat. It should be noted that a considerable amount of sand particles tend to accumulate around the one end of the equipment compartment for a long time due to the influence of the negative pressure area. Still, some windblown sand flows through the grille at the other end, then enters through second-end and leaves through adjacent grilles, forming a "shortcut".

**Figure 18:**
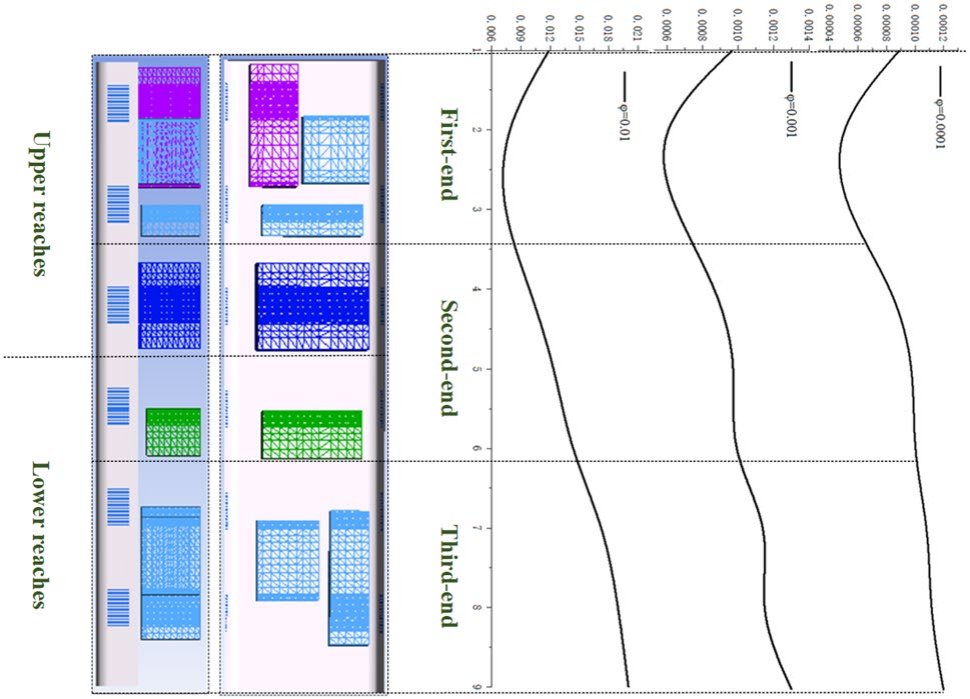
The average concentration curve within the equipment compartment for each concentration condition.

The flow field distribution under the four concentrations is basically the same, and the vortex structures of the flow are also similar. This indicates that the changes in particulate matter concentration have little effect on the wind speed distribution within the equipment compartment. However, when *φ* = 0, the flow velocity is relatively high, while at *φ* > 0, the overall flow velocity is lower than that of the no-sand wind condition, and as *φ* increases, the flow velocity becomes lower, the vortex structures become more obvious, and the sand retention phenomenon becomes more significant, as shown in Fig. 18. Therefore, as a wind-sand flow formed by mixing particulate matter with natural wind, the sand particles have distinct flow characteristics that differ from those of a no-sand wind flow, which is more consistent with the practical environmental conditions within the tunnel space.

### Temperature field in the equipment cabin

The flow field characteristics of no-sand wind and wind-sand flow were analyzed for the HWS-LT scenario above. Below, the temperature field and equipment heat dissipation characteristics inside the compartment under non-steady state conditions will be further analyzed.

**Figure 19:**
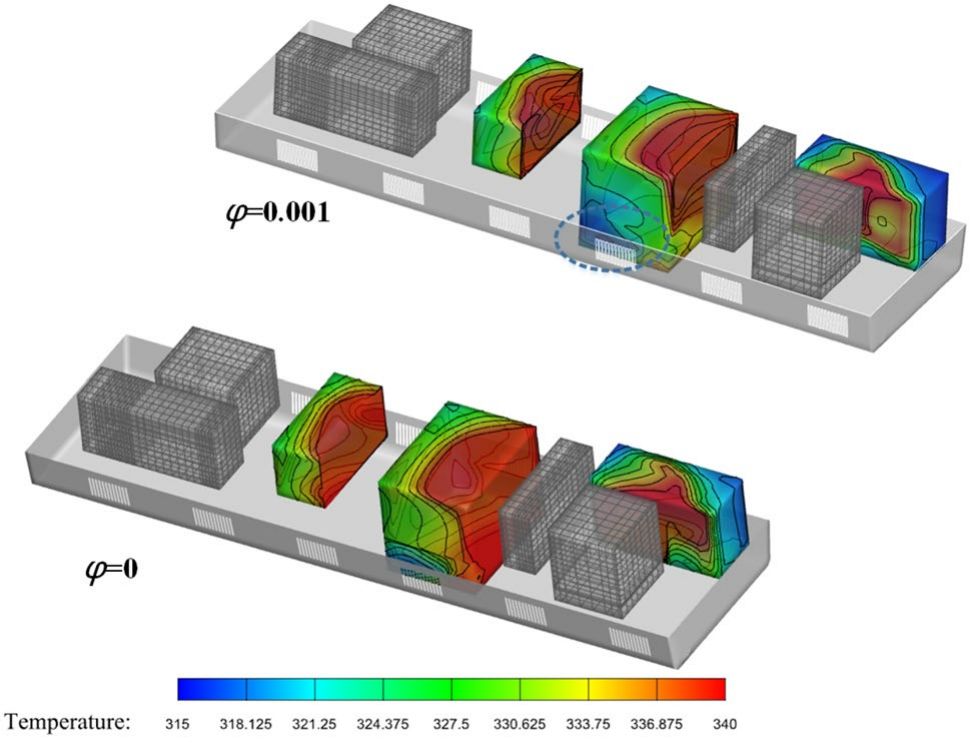
Temperature contour map.

In Fig. 19 I and II show the surface temperature distribution of the heating equipment inside the compartment under two different conditions. The surface temperature of the equipment is relatively high at both *φ* = 0 and *φ* = 0.004. At *φ* = 0, the local highest temperature distribution is relatively concentrated, with the highest temperature reaching 352 K (78.85 ℃), while at the same moment under the condition of *φ* = 0.004, the temperature at the measuring point is 346 K. The high-temperature areas are mostly distributed in the swirling recirculation areas, while the local temperature near the grille rapidly decreases because of the wind-sand flow with larger flow rates passing through it. It should be noted that there are significant differences in the heat dissipation effectiveness between the wind-sand flow and the no-sand wind. Previous studies on the heat dissipation in train tunnels are relatively conservative compared to actual engineering conditions, and the wind-sand flow seems to be more effective in reducing the surface temperature of the heat source, similar to the study of Song^36^. To observe this phenomenon more specifically, we adjusted the concentration of the wind-sand flow.

**Figure 20:**
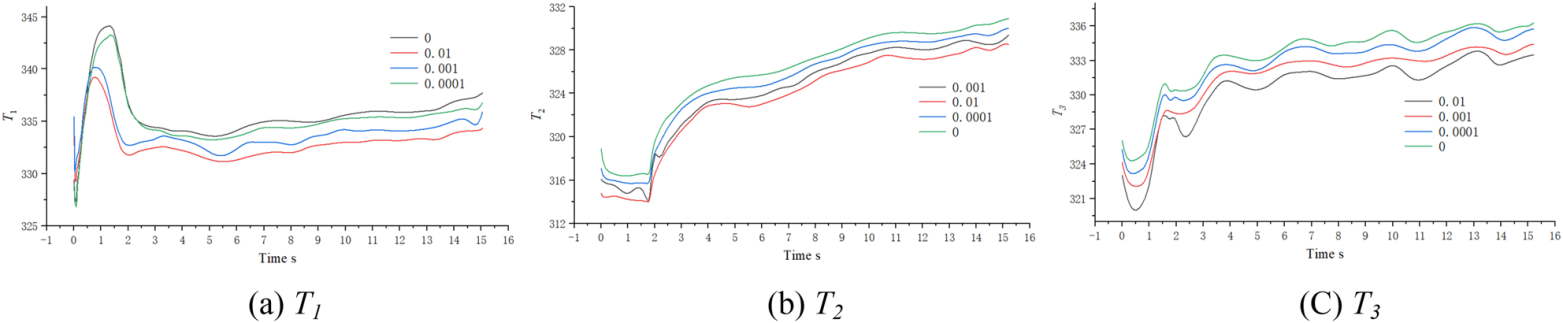
The diagram of temperature change with time, (**a**) T1, (b) T2, (**c**) T3.

The surface average temperatures of the equipment under the four concentration conditions are shown in Table 3. The ‘Temperature at the time of entering the tunnel’ *T*_*0*_ is not the temperature at the moment when the HST travels for 1.89 s, but the lowest temperature within the neighborhood of 1.89 s (± 0.3 s); ‘Temperature as you exit the tunnel’ *T*_*s*_ is the temperature when the HST travels for 15.02 s; ‘Mean temperature’ $\overline{T}$ is the average temperature during the tunnel journey; ‘Temperature difference’ $\Delta T = T_{s} - T_{0}$; ‘Climbing rate’ *r* represents the change in surface temperature of the heating equipment per unit time, $r = \Delta T/s$; ‘Heat-sinking capability’ *Q* represents the cooling benefit of the coolant (wind-sand flow), $Q = \frac{{\overline{T}_{{x^{\prime}}} - \overline{T}_{x} }}{{\overline{T}_{x} - 273.15}}$, where $\overline{{T_{{x^{\prime}}} }}$ represents the average temperature of the equipment numbered *x* at *φ* = 0.01, and $\overline{{T_{x} }}$ represents the average temperature of the same equipment at *φ* = 0. 273.15 K is the 0 ℃ in Celsius, and it should be noted that *Q* is affected by various factors, such as the assembly position of the equipment and the ventilation rate per unit time. Figure 20 shows the surface average temperature of the equipment inside the compartment changing with time when the HST passes through the tunnel.

**Table 3:** Surface temperature of the heat production equipment.

φ			0	1 × 10^–4^	1 × 10^–3^	1 × 10^–2^	Q
T_1_	T_0_	K	335.729	335.176	332.710	331.765	4.317%
	T_s_		337.718	336.754	335.873	334.328	
	$\overline{T}$		335.332	334.834	333.641	332.635	
	$\Delta T$		1.989	1.578	3.163	2.563	
	r	K/s	0.151	0.120	0.241	0.195	
T_2_	T_0_	K	319.128	318.241	317.089	316.180	3.995%
	T_s_		331.912	331.011	329.878	328.538	
	$\overline{T}$		327.081	326.283	325.349	324.926	
	$\Delta T$		12.732	12.77	12.789	12.358	
	r	K/s	0.969	0.972	0.974	0.941	
T_3_	T_0_	K	330.659	330.068	328.427	327.969	4.618%
	T_s_		336.266	335.744	334.420	333.482	
	$\overline{T}$		334.238	333.547	332.431	331.417	
	$\Delta T$		5.607	5.676	5.993	5.513	
	r	K/s	0.427	0.432	0.456	0.419

**Figure 21:**
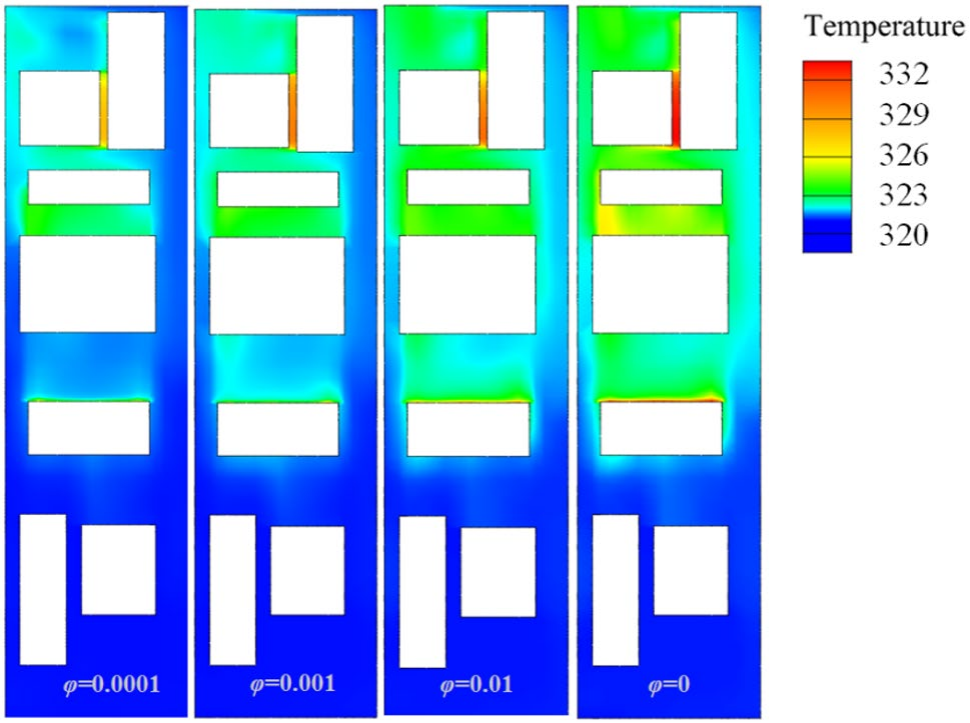
Horizontal section temperature field, Z = -0.9023.

**Figure 22:**
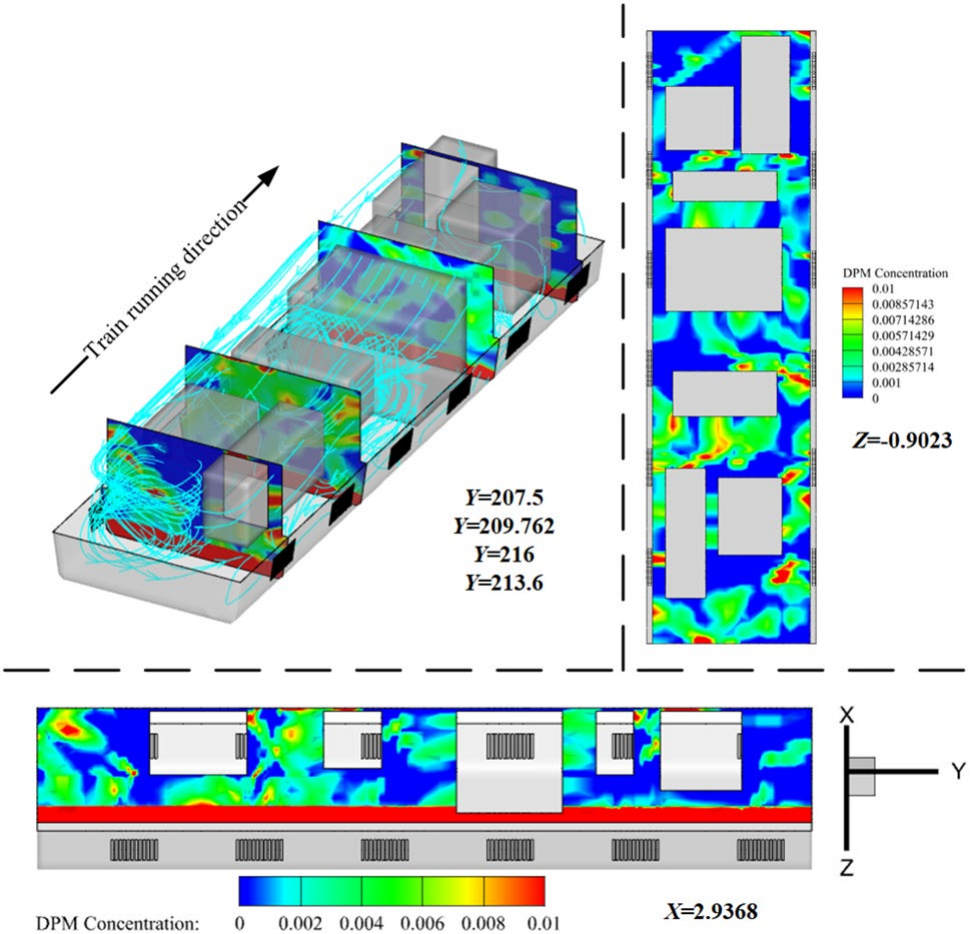
The concentration distribution map in the equipment cabin at 13 s.

**Figure 23:**
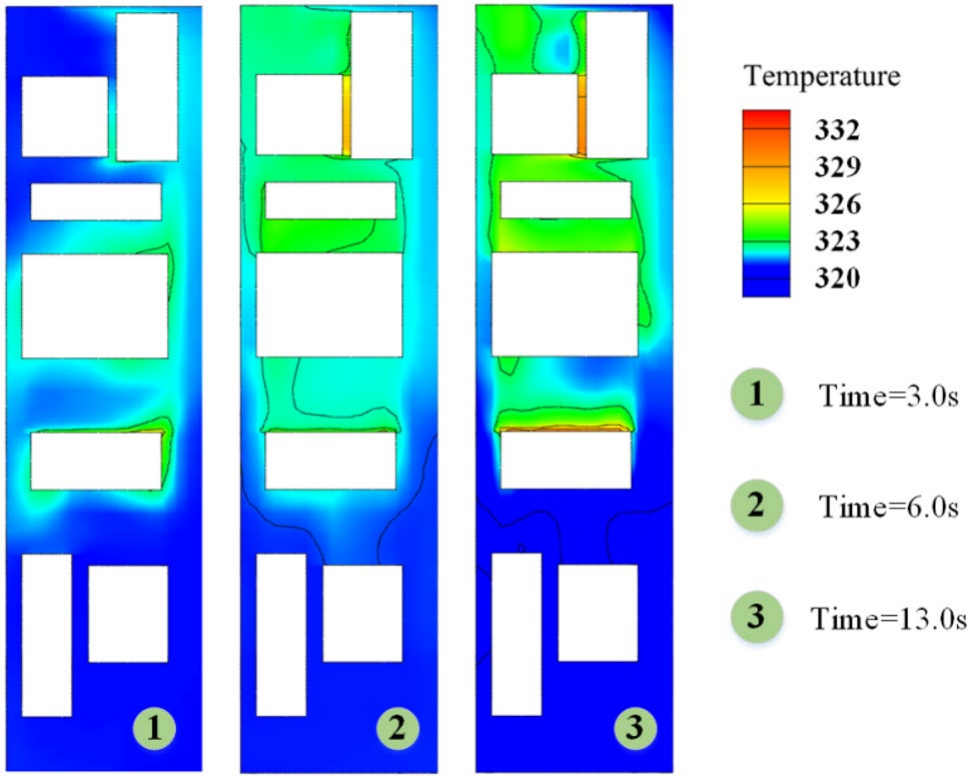
Horizontal section temperature field contour map.

As shown in Fig. 20, the temperature curves of the three groups of numbered curves all rapidly decrease at the initial moment, which is because the calculated scenario changes from steady state to non-steady state at 0 s, and the train is given a motion speed of 250 km/h, which causes the temperature to rapidly decrease in a short period of time. The temperature curves then sharply rise (a, b in Fig. 20), because the heat-generating components must find a balance point between heat release and heat generation in the numerical calculation process. Therefore, this study does not discuss the sharp increase or decrease part shown in Fig. 20. Based on Table 3 and Fig. 20, the temperature variations of the equipment under the four different conditions have the following characteristics:

- (1) After the HST enters the tunnel, the ventilation performance of the equipment compartment deteriorates. With the increasing time of train operation in the tunnel, the temperature of the equipment in the compartment, *T*_*x*_, gradually rises. At the same time, the repeated action of the tunnel pressure wave causes the pressure in the compartment to constantly change, and the wind-sand circulation inside the compartment is not stable, leading to the corresponding fluctuations of *T*_*x*_. The temperature of the compartment rises in a step-like manner as a whole. The temperature inside the compartment gradually accumulates in the form of heat energy, with ineffective heat dissipation. When the leading car reaches the tunnel exit, the temperature reaches its highest point. The air conditioner condenser has the highest temperature difference, with a temperature increase of 12.732 K.
- (2) During operation, *T*_1_ > *T*_3_ > *T*_2_, which is related to their own heat flux densities, and the rising trend of *T*_2_ is most obvious. The climbing rate *r*_2_ under the four different conditions is higher than 0.94 K/s. The rising trend of *T*_1_ is the slowest, *r*_1_ < 0.2 K/s, which is due to the high flow rate around the auxiliary inverter and the best ventilation effect, while small vortices are generated in the recirculation area where the air conditioner condenser is located, which affects heat dissipation, leading to more heat accumulation around the air conditioner condenser.
- (3) When *φ* = 0, it means that the air does not contain sand and dust. At this time, the average temperature of the temperature curve is the highest. As *φ* increases by orders of magnitude, *T*_*φ*_ decreases, indicating that the sand-containing wind has better heat transfer characteristics. The larger the *φ*, the more significant the decrease of *T*_*φ*_. Compared with natural wind, wind-sand flow (flow) with the same volume may be more conducive to heat dissipation. The *Q* value of the high-concentration wind-sand flow (1 × 10^–2^) is 4.317%, 3.995%, 4.618% respectively. It is worth noting that in Fig. 20(b), the temperature curve of *T*_2_ against *φ* shows a relatively insignificant cooling effect (*Q* < 0.04), which requires further analysis.
- According to Fig. 21, the temperature field in this horizontal section is unevenly distributed, with locally higher temperatures in the upstream area and lower temperatures in the downstream area. The highest temperature is distributed between the air conditioner condenser and low-power equipment 3 and 4. This is because most of the airflow in the equipment cabin forms a short circuit in the area near the compartment sidewalls, causing the wind-sand flow flowing from the second end to quickly flow towards the third end without producing recirculation. At the same time, some wind-sand flows past the sidewalls of the auxiliary inverter and traction inverter, taking away a considerable amount of heat. However, due to pressure effects, the flow rate at the first end grille is small, resulting in a much lower wind speed in the recirculation area of the first end than in the downstream area (as shown in I in Fig. 22). In addition, as shown in II in Fig. 22, the upstream wind-sand concentration is much lower than that downstream, and due to the dense arrangement of equipment in the upstream area, a small amount of sand enters the equipment compartment from the first end and collides with the surfaces of multiple devices to form eddies, resulting in the formation of a certain amount of particle aggregation (old sand) in the region. These sand particles differ from the wind-sand flow with lower temperatures. They are affected by the thermal radiation of equipment and convective heat transfer of gas phase, carrying and retaining high-temperature air with a low movement speed, ultimately reducing the cooling effect of wind-sand as a "coolant". Based on the above reasons, the air conditioner condenser has the most obvious temperature rising trend despite having the lowest heat flux density.
- Figure 23 shows the temperature contours of the horizontal end at three different time points, and the temperature inside the compartment is asymmetrically distributed. In the initial stage, the temperature on the right side is higher, and the heat gradually moves to the left after 4 s, and the temperature on the left side gradually rises, forming a sharp contrast with the temperature on the right side. With the evolution of time, the overall temperature gradually rises, and the heat accumulation in the upstream region of the equipment compartment is severe, with relatively insignificant contrast on both sides. According to the analysis of the velocity field and concentration field in Sect. 4.1, the main cause of this phenomenon is the asymmetrical intrusion of wind-sand flow into the compartment.

**Figure 24:**
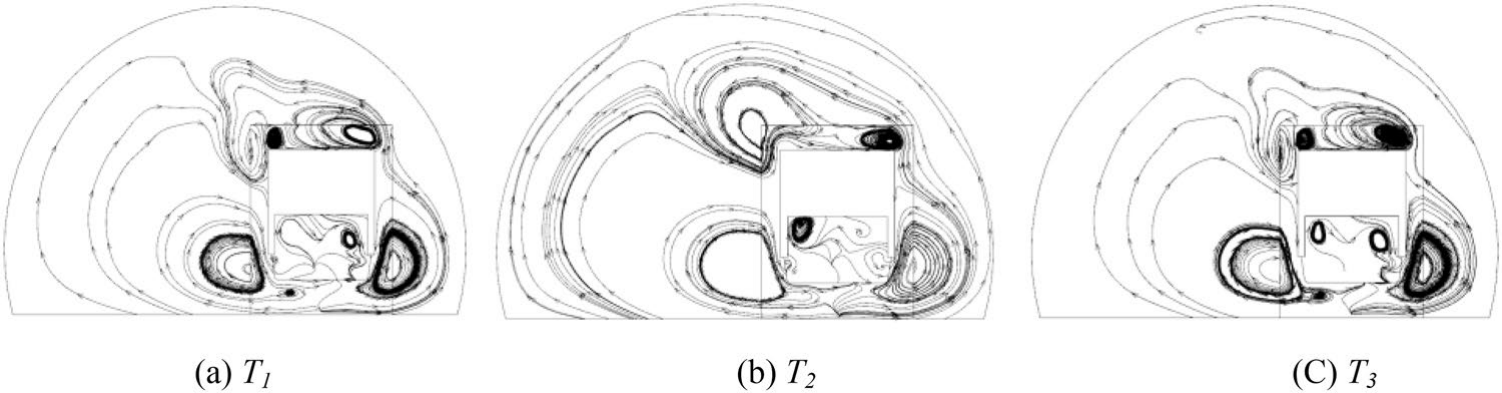
Vector streamline diagram, (**a**) T1, (**b**) T2, (**c**) T3.

Figure 24 shows the streamline distribution at *Y* = 231.6 m. The vortex structure in this area is more sensitive to the temperature field distribution, and the continuous vortex flow leads to a decrease in the efficiency of local air flow circulation and severe sand particle aggregation. Therefore, unlike in the absence of sand and dust, the asymmetric flow field has a significant impact on the equipment cooling in HST when driving in tunnels filled with wind and sand.

### Influencing mechanism analysis

**Figure 25:**
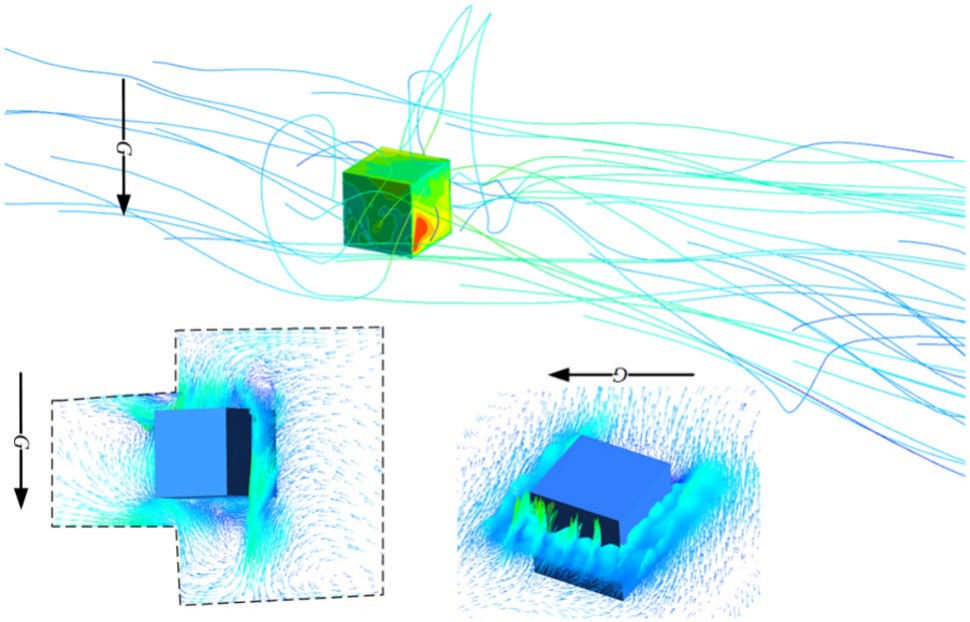
Flow field characteristic diagram of equipment block.

During the HST tunnel driving process, the flow field inside the equipment cabin evolves with time, but the flow velocity inside the compartment is generally within a certain threshold range. According to the analysis of the flow field inside the compartment under non-steady-state conditions in Sect. 4.1.3 of this paper, *v*_*eb*(max)_ ≤ 12 m/s, *v*_*eb*(min)_ ≥ 1 m/s. Therefore, for working conditions above 12 m/s and 1 m/s, this scenario is not considered. As shown in Fig. 25, the wind-sand flow released from the entrance exhibits laminar or turbulent flow during the transport process. When obstructed by equipment blocks and partitions, vortex or detached vortex structures accumulate, and finally flow out of the calculation domain through pressure outlets. The numerical simulation for the HWS-EB scenario can well reflect the flow field environment in which the heat-generating equipment is located in the HWS-LT scenario.

The specific values of the surface temperature of the equipment block determined by multiple variables are given in Table 4. For ease of description, we name and arrange the 7 working conditions in alphabetical order. The corresponding temperature variables are represented as: *T*_*a*_, *T*_*b*_, *T*_*c*_, *T*_*d*_, *T*_*e*_, *T*_*f*_, and *T*_*g*_.

**Table 4:** The average temperature on the surface of the equipment block.

Wind-sand flow velocity *v*_*eb*_/s	Temperature K						
	Roughness height *h* = 0.001 m					*h* = 0.002 m	*h* = 0.0005 m
	*D* = 0.3			*D* = 0.1	*D* = 0.001	*D* = 0.3	*D* = 0.3
	*φ* = 0.01	*φ* = 0.001	*φ* = 0.005	*φ* = 0.005	*φ* = 0.005	*φ* = 0.005	*φ* = 0.005
Working condition	a	b	c	d	e	f	g
1	311.636	315.511	312.794	313.397	316.011	312.708	312.882
2	311.606	315.079	312.585	313.012	315.579	312.457	312.601
3	311.425	315.029	312.178	312.589	315.129	312.101	312.538
4	311.418	313.886	312.125	312.504	314.360	312.056	312.496
5	311.410	313.201	312.076	312.387	313.902	311.935	312.304
6	311.423	312.654	311.798	312.150	313.241	311.651	312.005
7	311.266	312.285	311.538	311.888	312.975	311.514	311.779
8	311.261	312.027	311.492	311.685	312.627	311.397	311.672
9	311.139	311.687	311.412	311.524	312.087	311.306	311.580
10	311.052	311.588	311.254	311.311	311.688	311.132	311.332
11	310.997	311.424	311.158	311.213	311.524	311.064	311.215
12	310.919	311.275	311.066	311.125	311.355	311.018	311.134

**Figure 26:**
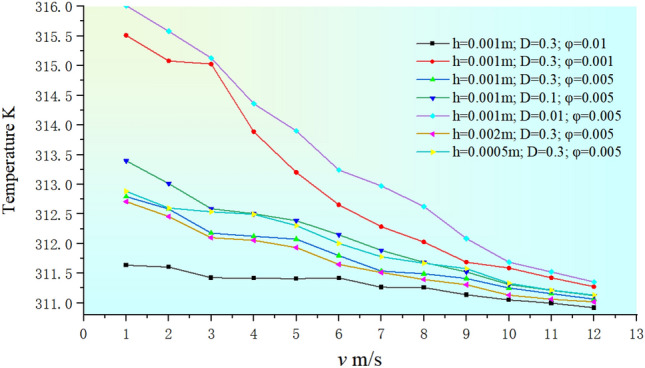
A line chart of the surface temperature.

In order to facilitate the analysis, we draw a temperature line chart determined by multiple variables based on the data in Table 4, as shown in Fig. 26.

(1) About the working condition of concentration: a., d., c.

a., d., c. working conditions are compared to show the temperature changes as a function of *φ’*. The overall temperature change trends under the three working conditions are similar, where the temperature decreases as the DPM concentration increases. This is consistent with the temperature variation of the heat-generating equipment in the HWS-LT scenario, which further validates the simulation results in Sect. 4.2. In addition, the mechanism behind this phenomenon can be preliminarily explained in combination with the previous description:

Due to the presence of sand particles, the mass and density of the mixture phase per unit volume are higher than those of a single gas phase (It must be emphasized that, for the purpose of facilitating the understanding and description of the thermodynamic characteristics of two-phase flow, the terms "mixture phase" and "average mixture characteristics" are purely qualitative.). Within a certain range of concentration (0 ≤ *φ’* < 0.01, ensuring that the mixture phase can still maintain its flow characteristics), an increase in *φ* leads to an increase in the density of the mixture phase per unit volume. Moreover, the specific heat capacity of sand particles is greater than that of air, so it can be regarded as an increase in the specific heat capacity per unit volume of the mixture phase. A higher specific heat capacity can take away part of the heat from the contact point between sand particles and equipment walls at the moment of contact. At the same time, as the concentration of the wind-sand flow increases, the frequency and contact area of particles with equipment walls also increase. Compared to a low-concentration wind-sand flow, a high-concentration wind-sand flow can quickly take away more heat. Secondly, unlike a single gas phase, the wind-sand flow increases the collision between particles during the transmission process, which makes heat transfer between particles more efficient. When collisions occur between particles, heat can be transmitted through the contact points between the particles. Under high concentration conditions, the contact area between particles increases, which increases the heat transfer path and promotes heat transfer and diffusion. This means that while the equipment block emits radiative energy outward (numerically), it also continually absorbs radiative energy emitted by the surrounding solid particles and converts it back into thermal energy. However, the temperature of the equipment block keeps rising and is naturally higher than the gas phase and discrete phase. Therefore, the result of the heat transfer is that the high-temperature equipment transfers heat to the low-temperature particles. Lastly, the presence of sand particles can indeed cause increased turbulence and disturbances in the motion of the discrete phase, leading to a higher possibility of heat transfer.

Due to the aforementioned effects, it is possible to more effectively transfer heat from the equipment to the particles, then from the particles to the surrounding environment. In the end, the wind-sand flow acts similar to a natural coolant, enhancing the heat dissipation effect, provided there is no continuous accumulation of sand particles. It must be emphasized that the above conclusions are valid in the premise condition established in this paper: there should not be prolonged accumulation of sand particles around the heat-generating equipment. According to Fig. 22, the equipment in the equipment cabin is all hanging equipment, and a small amount of sand accumulation on the bottom of the car does not affect the equipment. However, the wind-sand concentration in the recirculation area of the air conditioner condenser does not dissipate, and this wind-sand flow is not conducive to heat dissipation.

It is worth noting that *T*_*a*_ < *T*_*c*_ < *T*_*b*_ within a certain range, the higher the wind-sand concentration, the better the heat dissipation effect, especially at low speed where the advantage is more pronounced. However, the overall heat dissipation efficiency of low-speed wind-sand flows is poor, while high-speed wind-sand flows are relatively more advantageous. Moreover, although high-concentration working condition a. has better heat transfer characteristics, the temperature decrease is not significant during the process of *v*_*eb*_ changing from 3 m/s to 6 m/s. The temperature decrease is also not significant during the process of *v*_*eb*_ changing from 3 m/s to 5 m/s in working condition c., and similarly in working condition b. during the process of *v*_*eb*_ changing from 3 m/s to 4 m/s. The temperature curves of the three working conditions show a stepwise decrease, which is similar to the analysis of the heat-generating equipment in Sect. 4.2. Combined with the previous description, the mechanism behind these phenomena can be preliminarily explained:

Theoretically, there are some differences between high-speed wind-sand flow and low-speed wind-sand flows when they collide with equipment. These differences are mainly manifested as higher collision intensity, more collision frequency, more complex collision patterns, and possibly more dispersed sand particles in high-speed wind-sand flow. In terms of collision patterns, the airflow structure of high-speed wind-sand flow is more complex^42^. When the wind speed is low, the flow may be laminar instead of turbulent. Laminar and turbulent flows are two different states of gas or liquid flows^43–45^.

Secondly, high-speed wind-sand flow have higher flow speeds, and under the same concentration, the particles may collide with equipment more times per unit time, as they have faster speeds and shorter time intervals. Under the premise of completely elastic collisions, more collisions mean that sand particles are more likely to accumulate on the equipment surface, forming a thicker insulation layer. In contrast, the lower collision frequency of low-speed wind-sand flow means that the sand particles stay on the equipment surface for a relatively shorter time, resulting in a relatively thinner insulation layer, and making the wind-sand flow more effective in cooling. Therefore, the "Temperature change with concentration" is more pronounced at low wind speeds compared to high wind-sand conditions, while high wind speeds make the wind-sand flow more effective as a coolant.

(2) Conditions for Roughness height: c., f., g.

According to Table 4 and Fig. 26, *T*_*f*_ < *T*_*c*_ < *T*_*g*_, indicating that under constant surface roughness, increasing the Roughness height *h* is beneficial for heat dissipation. This result is consistent with the findings of Sheremet and Attar^46,47^. As the *h* increases, the height of the micro-roughness structure on the equipment surface increases, and the surface area correspondingly increases. At this time, the wind-sand flow is more likely to contact the surface, increasing the contact area between particles and the equipment surface. By increasing the contact area, the particles can more fully absorb the heat from the equipment surface and take away more heat, further verifying the previously explained principle of wind-sand heat dissipation. Secondly, an increase in *h* can also cause a more complex turbulent flow of wind-sand on the equipment surface, strengthening the heat transfer between particles and the equipment surface. In addition, turbulent flow can also disrupt the thermal boundary layer between particles and the equipment surface, allowing particles to more directly contact the equipment surface, thereby improving the efficiency of heat transfer.

(3) Working Conditions on Particle Diameter: c., d., e.

According to Table 4 and Fig. 26, *T*_*c*_ < *T*_*d*_ < *T*_*e*_, indicating that a decrease in sand particle size *D* is not conducive to equipment heat dissipation. Among them, Work condition e. (*D* = 0.001), and the particle size is at the nanometer level. Hatami et al. have proposed that nanometer-sized particle coolants have better efficiency in heat conduction. However, in this study, the equipment is relatively large in size, with higher surface roughness and roughness height. The use of nanometer-sized particles as coolants does not show significant results. On the other hand, millimeter-sized sand particles are more effective in taking away heat from the equipment surface. However, for sand particles of the same size (In the millimeter range), *T*_*c*_ < *T*_*d*_, which means that larger diameter sand particles usually have a larger volume, and can therefore provide more heat conduction paths. In contrast, smaller diameter sand particles have relatively limited heat conduction paths due to their smaller volume. In addition, as shown in Fig. 25, larger diameter wind-sand flow can cause more obvious air flow effects, which are less likely to form insulation layers, ultimately improving the efficiency of heat transfer.

In summary, within a certain concentration range, non-agglomerating high-speed wind-sand flows can serve as good coolants. When $0.0001 < D < 0.001$, larger diameter particles are more advantageous in taking away heat from the heat source surface. Similarly, under constant roughness, the more prominent the microstructure on the equipment surface (*h* +), the better the cooling effect of the wind-sand flow. Based on the above research, in order to more accurately reflect the heat dissipation influencing factors of the environment-heat source coupled system, and to provide feasible methods for the surface design of heat-producing equipment and environmental optimization, we have established an evaluation model (correlation equation) for the heat dissipation benefit of heat-producing equipment:

26 $$\left\{ \begin{gathered} \Delta = T - k_{a} \left( {\frac{\rho vl}{{\mu {\text{Re}}_{0} }}} \right)^{{k_{b} }} \cdot D^{{k_{D} }} \cdot h^{{k_{h} }} + \alpha_{k} \hfill \\ \rho = \rho_{air} + \left( {\rho_{DPM} - \rho_{air} } \right)^{\varphi } \hfill \\ {\text{Re}}_{0} = \frac{{\rho_{0} v_{0} l}}{\mu } \hfill \\ \end{gathered} \right.$$

where $\Delta$ represents the evaluation coefficient of heat dissipation efficiency, *T* represents the surface temperature of the equipment at the initial time, which can be either a fixed constant *T*_*0*_ or a variable represented by the heat flux density, i.e.,$T = \frac{{qL_{a}^{{}} }}{2\lambda } + T_{air}$, where *q* represents the heat flux or heat flux density of the equipment, in w/m^2^, *L*_*a*_ represents the wall thickness,$\lambda$ represents the thermal conductivity, *T*_air_ represents the ambient temperature, in K, and *ρ* represents the mixture density within the unit volume, kg/m^3^. where *α*_*k*_ represents the correction coefficient, which is used to measure temperature variations caused by other influencing factors. In Eq. (26), *α*_*k*_ is a variable representing temperature, and its specific value is determined through experimentation or fitting based on actual circumstances. The dimension of *α*_*k*_ is K. The specific value needs to be obtained by experiments or fitting according to the actual situation. *ρ*_air_ represents the density of the air phase, in kg/m^3^, *ρ*_DPM_ represents the density of the dispersed phase, in kg/m^3^, *Φ* represents the volume fraction of the dispersed phase in the mixed phase, Re_0_ represents the threshold at which the mixed phase transition from laminar flow to turbulent flow occurs, dimensionless, *ρ*_0_ and *v*_0_ represent the standard density and standard velocity corresponding to the threshold Re_0_, respectively. *k*_*a*_, *k*_*b*_, *k*_*D*_, and *k*_*h*_ represent the correction coefficients of the corresponding variables, and the specific values need to be obtained by experiments or fitting according to the actual situation. According to the actual physical meanings of *k*_*b*_, *k*_*D*_, and *k*_*h*_, it can be understood that they are exponents of respective terms, representing the linear or nonlinear changes in temperature when *D*, *h*, and fluid properties *ρ* and *v* change. Therefore, *k*_*b*_, *k*_*D*_, and *k*_*h*_ are dimensionless. Based on dimensional analysis, it can be concluded that *k*_*a*_ is a temperature correction coefficient. In addition, the validity of Eq. (26) must first satisfy the constraints of Eq. (14).

27 $$\left\{ \begin{gathered} C_{DPM}^{{}} > C_{air} \hfill \\ 0.0001 < D < 0.001 \hfill \\ \end{gathered} \right.$$

where *C*_*DPM*_ and *C*_air_ represent the specific heat capacity of the dispersed phase and air, respectively, in J/(kg·K). When the evaluation model (Eq. (13)) satisfies the constraint of Eq. (14),$\Delta \le 0$, and the larger $\left| \Delta \right|$ is, the lower the surface temperature of the equipment, indicating that the cooling effect of the wind-sand flow is better.

At a given time *t*_0_, the device surface temperature *T*(*t*) = *T*(*t*_*0*_), under the condition that the formula (27) is satisfied and the other influencing factors measured by *α*_*k*_ remain unchanged (That is, other influencing factors alone will not cause the temperature change at the next moment), So for the next moment *t*_0_ + $\Delta t$_,_ *T*(*t*_0_ + $\Delta t$) < *T*(*t*_*0*_) sufficient condition is $\Delta \le 0$_;_ For $\Delta$ the known basic variables are included *k*_*b*_ ≥ 1, *k*_*D*_ ≥ 1, *k*_*h*_ ≥ 1, therefore, $\left( {\frac{\rho vl}{{\mu {\text{Re}}_{0} }}} \right)^{{k_{b} }}$, $D^{{k_{D} }}$, $h^{{k_{h} }}$ all three are not completely monotonically increasing, *k*_*a*_ is only related to the *k*_*D*_, *k*_*h*_ , when evaluating the heat dissipation capacity, the value does not need to be specific. Because for the moment *T*(*t*_0_ + $\Delta t$) and *T*(*t*_*0*_) all have α(*t*_0_ + $\Delta t$) = α(*t*_*0*_). In addition, if there are two working conditions (working condition 1 has *D*_1_, *v*_1_, *h*_*1*_, *ρ*_1_; working condition 2 has *D*_2_, *v*_2_, *h*_2_, *ρ*_2_), $D_{1} \le D_{2}$_,_ *v*, *h*, *ρ* equal will be there *T*_2_(*t*_0_) < *T*_1_(*t*_*0*_). It can be further extended to include four-dimensional functions with four independent variables $\phi \left( {v,h,\rho ,D} \right)$_._ If there is $D_{1} \le D_{2}$_,_ and $\frac{\partial \phi }{{\partial D}} > 0$(In D, for example, the other three features are the same), there must be *T*(*D*_2_) < *T*(*D*_1_).

Applying this model to analyze the temperature variations of train heat-generating equipment can provide theoretical basis and methods for optimizing heat dissipation of the equipment. This indirectly improves the operational efficiency and safety of the train equipment compartment. In addition, this model can be used for optimizing the heat dissipation design of heat-generating equipment and evaluating the risks of the equipment under specific sandstorm conditions. It is important to note that this evaluation model relies on specific environmental conditions. Therefore, when there are uncertainties in the environmental parameters and heat source conditions, the model should be adjusted based on actual circumstances. It should be clarified that this model is an empirical pre-assessment model and does not fully quantify temperature fluctuations. Furthermore, the judgment criteria Δ is essentially the partial derivative of the function $\phi \left( {v,h,\rho ,D} \right)$ with respect to a single variable. Therefore, Δ can only assess the response changes when a single variable is varied and cannot measure the response changes under the combined effect of multiple variables.

## Conclusion

In order to clarify the influence of the high wind-sand environment in the long tunnel on the heat dissipation of the HST suspension equipment, this study selected a long tunnel (*W*_*t*_≈2.5 km) located in the second double-track of the Lanxin Railway, which is located in the "Qijiao Jing" wind area. Numerical studies were conducted using CFD methods for flow characteristics and heat transfer characteristics, and the following conclusions were drawn:

1. The 3D-DDES model can be applied well to the analysis of aerodynamic characteristics such as wind-sand particles and HST flow around, as well as convective heat transfer analysis. The application of this model in the secondary development of CFD numerical calculation can enhance the analysis of sand particle motion mechanisms.
2. Due to the relatively closed space environment in the tunnel, the surface temperature of the heat-producing equipment of HST continues to rise when it travels in the long tunnel, and the tunnel environment is not conducive to the heat dissipation of the equipment. Both non-sandy wind flow and wind-sand flow are affected by the asymmetric layout of the tunnel and exhibit asymmetric intrusion into the equipment compartment. When the inlet concentration *φ* increases (Including the "*φ* = 0" condition without sand), the sand particles are affected by vortices and tunnel walls, and the asymmetric flow of the wind-sand mixture becomes more obvious. The flow velocity of the mixed phase in the external flow field, the asymmetric phenomenon of the pressure on both sides of the equipment cabin (the pressure on the L side is higher than that on the R side), the inlet velocity, and the overall flow velocity in the compartment are all negatively correlated with *φ*. In addition, taking *φ* = 0.004 as an example, some sand particles in the compartment will form blockages at the grid or filter, and the outlet *ρ*_*Q*_ is smaller than the inlet *ρ*_*Q*_. The accumulation of dust and sand in the compartment during the entire HST journey is *m*_*s*_ = 321.63 g, indicating that the sand particles continuously accumulate in the equipment compartment, and the local concentration distribution is uneven, resulting in a significant upward trend in the surface temperature of some equipment. The temperature of the air conditioning condenser rises the fastest, with *r*_2_ > 0.94 K/s.
3. In the HWS-LT scenario, as the inflow concentration *φ* of the computational domain increases, the wind-sand flow from outside the compartment can better dissipate heat from the heat-producing equipment. Higher concentration wind-sand flow appears to be a better "coolant" (*Q*_*x*_ > 3.8%). Moreover, downstream equipment has a lower overall temperature, indicating that the cooling effect of the wind-sand flow is better (*Q*_3_ > *Q*_1_ > 4.0%). We used the HWS-EB scenario to verify this phenomenon by changing the surface geometric factors (*D* and *h*) of the heat transfer medium, fluid flow velocity *v*, and inflow concentration *φ*. One interesting conclusion is that a non-aggregating high-speed wind-sand flow can serve as a good coolant in a certain concentration range. Therefore, the superior heat dissipation of the downstream equipment can be explained: "short circuits" can easily form between the downstream grids, and the wind-sand flow has a higher flow velocity and more complex flow patterns, allowing more wind-sand volume to pass through the unit time. Furthermore, because wind-sand flow has a higher specific heat capacity at constant pressure *c*_*p*_, sand particles can take away some heat at the moment when they come into contact with the equipment walls, and the higher the concentration and flow velocity, the more frequent the particle–wall contact and the larger the contact area (*D* + , *h* +), resulting in a wider range of heat transfer pathways and better heat dissipation. Based on this, a heat dissipation benefit evaluation model was established for wind-sand flow. When $\Delta \le 0$, a larger $\left| \Delta \right|$ value means lower surface temperature of the equipment, proving that the cooling effect of wind-sand flow is better. The model describes the pre-evaluation mechanism of the heat dissipation situation of the "environment-heat source" system based on multiple determining factors.
4. In conclusion, this study has demonstrated the adverse effects of the HWS-LT scenario on heat dissipation of the train equipment and explained the heat transfer mechanism of wind-sand flow that differs from non-sandy wind from a mechanistic perspective. This study focuses on the motion behavior of sand particles in special scenarios and whether the involvement of particles can cause differences in heat transfer. It emphasizes the importance of sandstorm flow (two-phase flow) in equipment cooling and heat dissipation. It provides a theoretical basis and method for optimizing equipment heat dissipation, thereby improving the operational efficiency and safety of the train equipment compartment.

## Data Availability

The datasets generated during and analysed during the current study are available from the corresponding author on reasonable request.

## List of symbol

*g*: Gravitational acceleration
*ξ*: Normally distributed random number
*T*_*k*_: Air temperature
*T*_*l*_: Initial temperature of the particles
*T*_*vap*_: Particle volatilization temperature
*L*: Train length
*H*: Train height
*W*: Train width
*Q*_*1*_, *Q*_*2*_*, Q*_*3*_: Flux density
*E*: Fluid energy
*i*: Internal energy
*P*: Potential energy
*C*_*p*_: Specific heat capacity
*T*: Temperature
*m*_*p*_: Mass of the particle
*c*_*l*_: Heat capacity of the particle
*T*_*k*_: Local temperature of the continuous
*ε*_*p*_: Particle emissivity
*s*: Path length
*α*: Absorption coefficient
*n*: Refractive coefficient
*v*_*eb*_: Water intake flow velocity
*m*_*s*_: Sand accumulation
*T*_1_: Temperature of the auxiliary converter
*Q*: Cooling benefit
*q*: Flux density
*S*: Cross-sectional area
*L*_*t*_: Tunnel length
*h*_*a*_: Roughness height of equipment surface
*W*_*t*_: Tunnel width
*H*_*t*_: Tunnel height
C: Inlet
B: Outer
A: Wall
*u*_*y*_: Wind speed at height*y*
*u**'*: Friction velocity
k: Karman constant
*y*_*0*_: Aerodynamic roughness
*f*_*d*_: Delay-function
*λ*: Thermal conductivity
*S*_*T*_: Customized volumetric heat source
*F*_*D*_: Drag coefficient
*h*_*g*_: Convective heat transfer continuous phase
*σ*: Stefan-Boltzmann constant
θ_s_: Radiation temperature
*G*: Incident radiation
σ_s_: Scattering coefficient
*T*_*s*_: Local temperature
qr: Radiative heat flux
αed: Incidence angl
*ρ*_*Q*_: Grille mass flow
*T*_2_: Temperature of the auxiliary converter
*T*_*a*__~__*g*_: Temperature variable
*L*_*a*_: Wall thickness
*u*_*i*_: Velocity field
*ρ*: Air density
*r*_*a*_: Equipment surface roughness
*t*: Time
*p*: Pressure
*μ*: Dynamic viscosity
$$\tilde{u}$$: Filtered velocity
$$\tilde{p}$$: Filtered pressure
$\sigma_{ij}$: Stress tensor caused
$\tau_{ij}$: Subgrid-scale stress
*k*: Turbulence Kinetic Energy
*ε*: Turbulent Dissipation
*u*_*p*_: Velocities of the sand particles
*u*_*a*_: Velocities of the air
$\rho_{p}$: Sand particles density
*F*_*a*_: Additional force
*I*: Ten radiation intensity
*Ω*: Solid angle
$k_{\infty }$: Thermal conductivity of the continuous
Red: Reynolds number d
Ep: Equivalent radiation of the particle
Αp: Absorption coefficient of the particle
C: Anisotropic phase function
φ’: DPM concentration of HWS-EB
$$\overline{T}$$: Average temperature
*T*_3_: Temperature of the traction gearbox
∆: Evaluation coefficient
*Φ*: Dispersed phase in the mixed phase
*μ*_*t*_: Turbulent Viscosity
*v*_*t*_: Kinematic Viscosity
*h*: Roughness height
σ_k_, σ_ε_: Turbulent Prandtl numbers
*G*_*k*_: Mean velocity gradients
*G*_*b*_: Buoyancy effects
C_1_, C_2_, C_3_: Constants with different numerical values
*C*_*des*_: Model constant
$\Delta_{\max }$: Local maximum mesh spacing
*C*_*D*_: Drag coefficient of coupling model
$\mathop {m_{p} }\limits^{ \bullet }$: Particle mass flow rate
$$\Delta t$$: Time step
*F*_*other*_: Other interphase forces
Pr: Prandtl number
$$\overrightarrow {s}$$: Direction vector
$$\overrightarrow {r}$$: Position vector
$\overrightarrow {{s^{\prime}}}$: Heat dissipation vector
$$\rho_{w}$$: Wall emissivit
*V*: Running speed of the HST
*φ*: Volume fraction of sand
*φ*_0_: Initial concentration
*T*_s_: Temperature as you exit the tunnel
*T*_0_: Temperature at the time of entering the tunnel
*T*_air_: Ambient temperature
*l*: Characteristic length
